# Review on Adhesives and Surface Treatments for Structural Applications: Recent Developments on Sustainability and Implementation for Metal and Composite Substrates

**DOI:** 10.3390/ma13245590

**Published:** 2020-12-08

**Authors:** Ana C. Marques, Alexandra Mocanu, Nataša Z. Tomić, Sebastian Balos, Elisabeth Stammen, Asa Lundevall, Shoshan T. Abrahami, Roman Günther, John M. M. de Kok, Sofia Teixeira de Freitas

**Affiliations:** 1 CERENA, DEQ, Instituto Superior Técnico, Universidade de Lisboa, Avenida Rovisco Pais, 1049-001 Lisboa, Portugal; ana.marques@tecnico.ulisboa.pt; 2 Faculty of Applied Chemistry and Materials Science, University POLITEHNICA of Bucharest, 1-7 Gh. Polizu, 011061 Bucharest, Romania; alexandra.mocanu@upb.ro; 3 Innovation Center of Faculty of Technology and Metallurgy, Belgrade Ltd., Karnegijeva 4, 11000 Belgrade, Serbia; ntomic@tmf.bg.ac.rs; 4 Department of Production Engineering, Faculty of Technical Sciences, University of Novi Sad, Trg Dositeja Obradovića 6, 21000 Novi Sad, Serbia; sebab@uns.ac.rs; 5 Department Adhesive Bonding, Institute of Joining and Welding, Technische Universität Braunschweig, Langer Kamp 8, D-38106 Braunschweig, Germany; e.stammen@tu-braunschweig.de; 6 RISE IVF AB, Lindholmspiren 7 A, 417 56 Göteborg, Sweden; asa.lundevall@ri.se; 7 Research Group Electrochemical and Surface Engineering (SURF), Department of Materials and Chemistry, Vrije Universiteit Brussel, Pleinlaan 2, B-1050 Brussels, Belgium; shoshan.abrahami@vub.be; 8 Laboratory of Adhesives and Polymer Materials, Institute of Materials and Process Engineering, Zurich University of Applied Sciences, Technikumstrasse 9, 8401 Winterthur, Switzerland; roman.guenther@zhaw.ch; 9 Laboratory for Multifunctional Materials, Department of Materials, ETH Zurich, Vladimir-Prelog-Weg 5, 8093 Zurich, Switzerland; 10 GKN Fokker Aerostructures BV, Industrieweg 4, 3351 LB Papendrecht, The Netherlands; john.dekok@fokker.com; 11 Faculty of Aerospace Engineering, Delft University of Technology, Kluyverweg 1, 2629 HS Delft, The Netherlands

**Keywords:** adhesive, surface pre-treatments, sustainability, certification

## Abstract

Using adhesives for connection technology has many benefits. It is cost-efficient, fast, and allows homogeneous stress distribution between the bonded surfaces. This paper gives an overview on the current state of knowledge regarding the technologically important area of adhesive materials, as well as on emergent related technologies. It is expected to fill some of the technological gaps between the existing literature and industrial reality, by focusing at opportunities and challenges in the adhesives sector, on sustainable and eco-friendly chemistries that enable bio-derived adhesives, recycling and debonding, as well as giving a brief overview on the surface treatment approaches involved in the adhesive application process, with major focus on metal and polymer matrix composites. Finally, some thoughts on the connection between research and development (R&D) efforts, industry standards and regulatory aspects are given. It contributes to bridge the gap between industry and research institutes/academy. Examples from the aeronautics industry are often used since many technological advances in this industry are innovation precursors for other industries. This paper is mainly addressed to chemists, materials scientists, materials engineers, and decision-makers.

## 1. Introduction

### 1.1. Overview

Adhesively-bonded joints (ABJs) consist of generally two joined components and the layer of adhesive between them [1,2]. The most important part of the ABJ is the adhesive itself, that is, the component depending mainly of the formulation, followed by the preparation process that precedes the application of the adhesive. Adhesive bonding requires a special manufacturing process, which involves three main aspects: qualified methods/processes, trained operators, and dedicated tools.

Complex and advanced technologies, or series of technologies, have arisen to enable the application of adhesives in many fields. The diversity of substrates and the continuous development and introduction of new processes and materials has placed the adhesives technology as one of the most swiftly expanding manufacturing endeavors.

As in any emergent technology, strict environmental regulations, such as those emitted by the European Chemicals Agency (ECHA), including REACH (Registration, Evaluation and Authorization of Chemicals) compliance, play an important role, which dictates products certification and commercialization. These aspects are affecting the sector, driving a marked growth in consciousness to reduce fossil dependence and mitigate global pollution, which result in an increasing demand for products manufactured from renewable and sustainable sources.

As for the future of adhesives, since they are polymeric materials, the recommendations toward sustainability are: understanding their impact throughout product lifecycle, developing new sustainable polymeric materials, closing the loop of plastics recycling, and understanding and controlling plastic degradation [3]. Developments in chemistry will be critical to understanding and mitigating the impact of plastics in the environment.

This paper complements other reviews in the topic [4,5,6,7,8,9,10], by giving an overview of eco-friendly emergent adhesive technologies, surface treatments that precede the application of adhesives, new trends in adhesive waste management, including recycling and design for disassembly, and introduces new insights on the connection between Research & Development (R&D) efforts, industry standards and regulatory aspects, which unquestionably influence the roadmap of adhesives chemistry development.

The paper is divided into four main topics: Section 2 gives an overview of bio-based raw materials, polymer recycling, and the possibility to design “greener” formulations for structural adhesives using microencapsulation of hazardous but very efficient cross-linkers; Section 3 reviews how to address REACH regulations on the surface treatments required for adhesion; Section 4 addresses the existing and emerging technologies for debonding to allow recyclability and repair of bonded structures. Finally, Section 5 gives a critical overview on the certification/qualification process for the successful implementation of these new emerging technologies in real structures.

### 1.2. Well-Established Industrial Adhesives

The adhesive crucial role is to transfer the load from one base material to the other. To date, a wide array of adhesives found practical application and were tested experimentally, in order to find the most suitable way of creating ABJs between a wide spectrum of different engineering structural materials (substrates), ranging from metals and alloys, composites, to natural materials as different types of wood.

Adhesives and sealants can be classified in several ways: by chemical composition—adhesive binder, natural vs. synthetic, organic vs. inorganic, structural vs. nonstructural, curing or setting mechanism, etc. [11]. However, the most common classification is based on the adhesive binder, as listed in Table 1.

Moreover, different adhesive technologies can be categorized by increasing order of load bearing capability, typically from ca. 0.01 to 40 MPa of overlap shear strength, as follows: pressure sensitive adhesives, reclosable fasteners, contact and spray adhesives, acrylic foam tapes, hot melt adhesives, adhesive sealants based on polyurethane and hybrids, polyurethane adhesives (PUR), epoxy, and acrylic and urethane structural adhesives. Structural adhesives provide several advantages such as strong bonds, design flexibility and process efficiency. Well-established industrial adhesives encompass different chemistries, curing methods, open times and final bond capabilities, including low-odor and non-flammable versions of certain chemistries to meet specific regulatory and safety requirements.

### 1.3. Opportunities and Challenges in the Adhesives Sector

ABJs are commonly used in a wide variety of applications. Some estimations claim that over 20% of ABJ are used in construction (windows, doors, pipes, flooring, insulation, glazing, tiles, among others). However, other applications have emerged, such as those in the fields of electronics, energy, marine, automotive, and aerospace industries [2,10,14].

#### 1.3.1. Replacement of Mechanical Fasteners by Adhesives: A Reality?

Although adhesive bonding technology has shown potential for several decades in the aerospace industry, its application as a joining technology and assembly method frequently occurs in secondary parts of the aircraft structures. Typically, in primary structures, different parts of aluminum sheets, being the most common ones, AA2024-T3 and AA7075-T6 (bare and cladded materials), are assembled relying on fasteners, namely rivets. Indeed, recent developments in the automatization have made riveting economically attractive, however, this bonding technology exhibits some limitations.

Some of the advantages of ABJs vs. alternative joining techniques such as mechanical fastening methods as riveting, especially in aerospace applications are shown in Table 2.

As a consequence of the noted advantages of adhesives vs. other means of joining, the application of ABJs has grown significantly in recent decades, with a good prospect to become even more attractive in the future [12]. For instance, when joining composite components, mainly epoxy and polyurethane (PU) adhesives are used. They are characterized by high strength and stiffness values at high elongation levels. This results in very positive impact and fatigue properties of bonded joints.

Nowadays aircraft manufacturers are increasingly using carbon fiber-reinforced polymers (CFRP) as a lightweight and robust replacement of aluminum. Riveting such composite materials has a lot of hazards, such as fiber breaking, stress concentrations and black dust. Adhesive bonding is the most promising joining technology in terms of lightweight and performance for assembling composite parts. However, the status in the aircraft industry is that bonded joints without additional fasteners are only certified for assembling secondary structures of an aircraft, whose failure is not detrimental for aircraft safety. In comparison with metals, composites bring extra challenges, since the manufacturing processes use release agents, which can reduce adhesion strength, which cannot be predicted with the conventional non-destructive testing, such as ultrasound. New alternatives are urgently needed to use the full potential of bonded systems in composite aircraft structures. Efforts are being done in this sense, such as European collaborations, namely the COST project CERTBOND—“Reliable roadmap for certification of bonded primary aircraft structures” (certbond.eu), which addresses this need, by tackling the scientific challenges in the different stages of the life-cycle of a bonded structure.

Interfacial failure is a critical aspect that must be avoided during the in-service life of bonded structures since it is unpredictable. It is proved to be connected to the lack of chemical bonding between the bonded surfaces. It is hence believed that the next breakthrough in the adhesive bonding technology requires the fully integrated research and development, from the basic chemistry up to the final design and manufacturing. Only such a holistic development and suitable product design can provide the required reliability to be demonstrated for aerospace certifications.

#### 1.3.2. REACH Evolution and Its Impact on Adhesives Industry

Replacing hazardous chemicals and manufacturing processes by safer chemicals and greener technologies has been a key investment of adhesive companies, to avoid regrettable roadblocks in product commercialization in a near future. In addition to the benefits to companies, the environment, and the health of workers and consumers, this can also have a significant positive impact on the implementation of a circular economy. The continuous evolution of REACH regulation brings a critical challenge in what concerns requalification of new adhesive versions and upgrade of safety equipment and procedures. In some cases, it leads to product obsolescence and the stop of production lines if adequate measures are not duly implemented.

An example of REACH compliance is herein given for the case of bicomponent polyurethane formulations. Component B consists of 4,4′-methylenediphenyl diisocyanate (MDI), which, despite being considered less hazardous than other isocyanates (e.g., toluene diisocyanate—TDI) it is classified as Harmful (Xn) by ECHA, namely irritant for skin, eyes and respiratory organs. It is also suspected to cause cancer and severe damage to organs through prolonged exposure. According to Annex XVII of REACH (Council Directive 76/769/EEC of the European Union), the restrictions for MDI involve the use of this compound only by qualified personnel with no asthma, eczema, or allergic reactions to isocyanates, in well-ventilated areas and with appropriate protection equipment. In addition to selecting less hazardous isocyanates, companies, such as Henkel, have released improved formulations, such as TEROSON PU 6700 ME (MicroEmission technology) which is a solvent-free polyurethane bicomponent product, with very low volatile organic compounds (VOCs), with no R40 (limited evidence of a carcinogenic effect) risk phrase due to micro-emission property.

Another example of REACH impact on adhesives industry is for epoxy adhesive formulations, as some of the main current epoxy-based precursors are classified as H341, i.e., suspected of causing genetic defects, namely triglycidyl-*p*-aminophenol (TGPAP) and *N*,*N*′-tetraglycidyl diaminodiphenylmethane (TGDDM). On the other hand, amine-based precursors, such as 4,4′-diaminodiphenylsulfone (44DDS) are in the scrutiny for being possible endocrine disruptors (included in the Community rolling action plan, CoRAP) and trimellitic anhydride is classified as a substance of very high concern (SVHC). Moreover, widely employed organic solvents in these adhesives, such as n-methyl-2-pyrrolidone (NMP), are classified as H360, i.e., may damage fertility or the unborn child, are classified as SVHC and included in the Candidate list and restricted under Annex XVII of REACH. NMP’s production, use and sale are forbidden, as substance or as component of mixtures in concentration equal or greater than 0.3%, since May 2020 (Reg. 2018/588).

Another substance also quite common in adhesives formulations, mainly used for wood-based panels, is formaldehyde, which is carcinogenic, suspected to be mutagenic and skin sensitizer. It is restricted under Annex XVII of REACH (Reg. 2018/675) since 2018.

Inclusion in the Candidate List can trigger certain legal obligations for the importers, producers and suppliers of an article that contains such a substance. Thus, there is plenty of room for the identification and development of greener, non-hazardous chemicals that comply with safety and environment regulations and enable the required performance of adhesives.

#### 1.3.3. Adhesives from Renewable Resources

The production of the above-mentioned resins has relied on non-renewable petroleum resources. Due to the growing demand of environmentally friendly, green and sustainable materials, as well as economic and availability issues of petroleum resources, there has been a growing interest in the development of environmentally friendly adhesives from renewable resources. For the sake of clarity, a biopolymer is a naturally occurring polymer, such as cellulose or starch, while a bio-based or bio-derived plastic is a polymer that is produced from biological resources, including chemicals derived from plants and algae. e.g., polylactide is produced from sugar, which is harvested from plants like sugar cane [3].

Indeed, the first uses of bio-adhesives widely preceded the use of synthetic adhesives, since they date back from the middle and upper Paleolithic period [16]. Although the effectiveness of natural adhesives (biopolymer) was satisfactory for those times, they are all but efficient and well suited to modern production demands [17]. However, due to advances in polymer synthesis, several man-made, mass produced adhesives suitable for joining of different base materials have emerged and found practical application in ABJs. Currently, there is a wide variety of eco-friendly adhesives and sealants, based on cellulose, starch, lignin, vegetable oil, and protein-based silanes, which are yet mainly employed for non-structural applications. Investment and advances on biopolymer production and processing can lead to a decrease of manufacturing costs, while creating new “green” businesses opportunities [4,10].

## 2. Eco-Friendly Emergent Structural Adhesives

The desire for cleaner and healthier environments, and the evolving regulatory aspects in this direction, are promoting the replacement of petroleum-based raw materials with natural components, and, at the same time, limiting the use of non-renewable resources for adhesive formulations. The concept of the circular economy was created in order to optimize a more efficient consumption of products while making people more aware of the anthropogenic activities that are related to high levels of pollution and waste products [18]. From another perspective, bio-based materials have gained significant attention in almost all fields due to the well-known issues that regard the use of petroleum-based materials [4,19,20,21,22]. These trends are also noticed in the field of adhesives, where the production of monomers from bio-renewable resources and waste products have been comprehensively investigated. This section emphasizes the importance of bio-based raw materials, polymer recycling, and the possibility to design “greener” formulations for structural adhesives which contribute to efficient waste management, economic benefits, and environment/health protection.

### 2.1. Renewable/Bio-Derived Adhesives

Efforts have been made in adhesive technology toward the use of renewable materials to produce commercial counterparts with equal or even better performance. Using renewable resources in adhesive formulations is not, per se, necessary and sufficient reason for commercialization. Market penetration might be particularly difficult if associated additional costs are envisaged and if a straightforward drop-in substitution for the present technology is not provided. Moreover, it will depend if existing and new biopolymers companies are able to increasingly adopt highly efficient, continuous production technologies and if an effective channel of increased knowledge creation and transfer to the industry is created. Commercialization of bio-based adhesives has another benefit in terms of circular economy, regarding their straightforward reutilization since they are more prone to biodegradation. The presence of ester linkages, for instance, present in many biopolymers is known to promote biodegradability [23,24,25]. This chapter will show the advantages of adhesives derived from varied bio-sources, designed for wide spectrum of applications and opportunities.

#### 2.1.1. Utilization of Vegetable Oils

The advantage of vegetable oils, such as availability, low toxicity, low price, and biodegradability, among others, brought them in the spotlight of the chemical industry, having already achieved commercial use. Polymers and composites derived from vegetable oils are found in applications such as paints, coatings, and adhesives [26]. Adhesion properties of pressure-sensitive adhesives based on vegetable oils can be tuned by the introduction of various functional groups. As an example, an adhesive obtained from acrylate methyl oleate showed typical pressure-sensitive properties, exhibiting similar properties to commercial products [26]. Adhesive gels with tunable viscoelastic properties that have carboxyl groups as adhesion enhancers were synthesized by condensation of dimer fatty acid and diols together with maleinized triglycerides [27]. In general, the technological processes of obtaining monomers and polymers from vegetable oils were shown to be feasible and capable of replacing petroleum products. The actual trend in the field of plant oil-based polymers is to increase their share in the market with efforts to maintain or improve overall properties by developing new ones for special purposes.

In 2019, Patel et al. [28] presented the first completely bio-based two-component structural epoxy adhesive. In their own preliminary work, bio-based monomers of n-alkyl esters of diphenolic acid (DGEDP epoxides) with comparable thermal and mechanical properties to classical DGEBA epoxides [29,30], were developed. These were copolymerized with a derivative of cashew nutshell oil [31]. Nanocrystalline cellulose served as rheological auxiliary and bio-based bis (furfurylamine) as a cross-linking agent. An adhesive was obtained which shows a strength of 20 MPa on aluminum substrates and thus, a comparable strength to that of petroleum-based adhesives, found, e.g., in automotive bodies.

#### 2.1.2. Utilization of Wood Derivates (Lignin, Tannin, Cellulose)

Abundant bio-based raw materials like lignin, tannin, and cellulose comprise numerous hydroxyl groups which may act as a reactive source in the production of adhesives [32,33]. In addition, the high content of phenolic groups contributes to higher fire retardancy of the system.

Lignin is a byproduct of biorefinery processes that can be obtained in various forms, such as organosolv-, kraft-, sulfonate-, soda- and enzymatic hydrolysis lignin [34]. Mechanical and other desired properties may be achieved by modification of lignin by grafting reactions [35,36]. With the aim of replacing conventional bis-phenol A components in the epoxy resin systems, reactive epoxy functional groups are introduced into the lignin structure, establishing a renewable epoxy network [37,38,39]. Formaldehyde emissions in phenol-formaldehyde resin adhesives can be reduced by using lignocellulose ethanol residue, having already activated the lignin [40,41].

Another type of natural polyphenols found in woody plants are condensed tannins [41]. Some tannins (i.e., from plants quebracho and wattle) are used in the production of formaldehyde wood adhesives since the 1970s [42]. For the reduction of formaldehyde emission, new formaldehyde-free systems were developed due to the presence of catechol group in tannins that reacted with polyethyleneimine (PEI) with strong adhesion and water resistance [43].

Cellulose nanomaterials are most used in three forms: nanofibrils, nanocrystals (CNC), and bacterial cellulose. Using cellulose as a bio-renewable source, strong and biodegradable products of low weight can be obtained [44]. The cellulose nanofibrils as a sole binder in low-density particle board panels meets the industry requirements considering the mechanical properties.

Despite the desirable complex network apported by cellulose to the adhesive polymeric structures, its semi-crystalline nature limits dissolution and melting capability [45]. Nevertheless, several cellulose derivatives, such as trimethylsilylcellulose (TMSC) and sylilated cellulose compounds, have been synthesized with increased solubility in tetrahydrofuran, toluene or other common organic solvents targeting highly flexible biodegradable film-forming adhesives or sealants [46,47,48]. Cellulose nanofibrils modified with aminopropyl triethoxysilane (APTES) can crosslink to surfaces containing hydroxyl groups, potentially representing an adhesive effect to polar surfaces [10,49]. Water resistance of plywood joints can also be improved by CNC or acetylated CNC modified with soybean or acetylated soybean flour [50]. Moreover, the hydrolyzed soy protein isolate (HSPI) can be used in biodegradable urea-formaldehyde adhesives for bonding wood products [51]. Additionally, the incorporation of only 2 wt.% of surface modified CNC with 3-aminopropyltriethoxysilane (APTES) into urea-formaldehyde adhesive formulation [51,52] was found to lead to a significant improvement (30%) of the rupture strength and rigidity (modulus of elasticity) of Medium Density Fiberboard (MDF) type fiberboards [53]. Moreover, it was demonstrated that the presence of modified CNC with silane derivatives can seriously reduce formaldehyde emissions in the case of urea-formaldehyde adhesives [53]. Further study on modification, characterization and application in different adhesive systems is still in need for the commercialization of such technology by stakeholders.

An example of a well-established, commercialized primary building block derived from a renewable natural resource is cardanol, a natural phenolic material obtained by distilling cashew nutshell liquid (CNSL). CNSL can be found in the honeycomb structure of the cashew nutshell and is considered a by-product (non-food chain material) of the cashew nut industry. Cardanol is composed of an aromatic ring with a hydroxyl (OH) group and a long aliphatic side chain, which bring valuable intrinsic benefits to adhesive materials. The aromatic ring gives a strong chemical resistant backbone while the OH group provides high bond strength and good reactivity for a fast and low-temperature cure. The side chain provides excellent water resistance, good flexibility, low viscosity, and extended pot life. Cardolite Corporation manufactures and commercializes a wide range of specialty curing agents, resins, diluents for coatings, and adhesives, derived from cardanol. Most of them exhibit more that 65% of bio-content. The synthesis of cardanol building blocks to polymer synthesis is described in the literature [54].

#### 2.1.3. Utilization of Polysaccharides

Potato starch can be used in transesterification reactions with natural oils to prepare polyester polyols for the use in the manufacturing of PU adhesives, namely from the reaction of polyester polyols and an aromatic adduct based on TDI [55]. Such obtained bio-PU wood adhesives showed superior properties when compared to the commercial products. Chitosan is a polysaccharide obtained after deacetylation of chitin that is found mostly in crustaceans, insects, and fungi [56]. Chitosan is used as a biomedical adhesive due to its hemostatic properties [57], wood adhesive [58,59], and adhesive for metallic surfaces [60]. By analyzing these adhesives properties, an opportunity in the competitiveness with petroleum counterparts might be in place.

#### 2.1.4. The Role of Nanoparticles in Bio-Based and Commercial Adhesive Formulations

Certainly, one of the greatest challenges in eco-friendly adhesives is the poor resistance to high humidity. As stated in Section 2.1.2, some strategies have been explored to increase water resistance of adhesives by adding modified CNC, but different approaches are worthy of being mentioned. The addition of inorganic layered clays or nanoparticles into different adhesive formulations appears as a good approach to improve the water resistance of bio-based adhesives, while keeping or sometimes increasing their mechanical properties as well [61].

For instance, starch-based wood adhesives registered a 90% increase in shear strength in a wet state by adding 1 wt.% Montmorillonite (MMT) compared to the unmodified adhesive. Similar results were obtained by adding 4 wt.% TiO_2_ nanoparticles in starch-modified vinyl acetate wood adhesive formulations [62]. Additionally, some research studies were focused to improve the performance of commercial phenol-formaldehyde resins by adding lignin nanoparticles to the adhesive wood formulations. The mechanical tests revealed that only 5 wt.% of lignin nanoparticles (by resin) registered higher shear strength compared to neat adhesive [63]. It was also shown that depolymerized lignin stimulated the hydrophobization of camelina protein for wood adhesives; after a three-cycle water soaking procedure of the wood panels a five-times increase of the wet shear strength was registered (from 0.28 to 1.43 MPa) [64].

However, epoxy adhesives, as probably the most widely used adhesives, were shown to be the most popular base for modification with nanoparticles. In studies done by Saraç et al. [65,66], three types of nanoparticles were added to the basic epoxy adhesive: Al_2_O_3_, TiO_2_ and SiO_2_ (15–20 nm size) at three loadings, 2, 4, and 6 wt.%, for joining AISI 304 austenitic stainless steel. It was demonstrated that nanoparticle addition was found to be beneficial for improving the shear strength of the ABJs. Similar results were obtained in the case of Araldite F epoxy resin cured with cycloaliphatic secondary amine that was modified with nanosized SiO_2_ particles (20 nm size) in order to improve the tensile strength of unspecified type of stainless-steel plates [67]. Thus, at concentrations of 10 and 20 wt%, not only the ABJ was improved by 20%, but also the substrate wettability was increased [67]. Shear strength, tensile strength, and elongation at break of dissimilar bonded surfaces like steel and carbon-fiber-reinforced plastic (CFRP) were also improved by adding nanosilica up to 1 wt.% [68].

Nevertheless, in all cases, the overloading of the adhesive formulations with nanoparticles can induce undesirable effects in the adhesion performance [67,69]. Although size and type of the nanoparticles are important characteristics for adhesive bonding, dispersion of the nanoparticles in the adhesive matrix is a crucial factor since the appearance of agglomerates and aggregates can cause premature failure of adhesive joints. That means, after the crack propagates and reaches agglomerates, they fracture, transmitting the stress to the matrix suddenly, overloading it, and leading to drastic drop of mechanical properties [70,71]. Zeta potential measurements for nanoparticles distribution [72,73], scanning electron microscopy (SEM), and atomic force microscopy (AFM) could be useful analyses to determine the size distribution of the nanoparticles, the aggregate areas, or the roughness of the film-forming materials.

Although these methods provide some information about the morphology of the adhesive layer, they are not enough to predict the mechanical properties of the adhesive. In some nanoparticles containing adhesives, contact angle measurements were used to determine the surface tension of the adhesive layers to indicate if there is a correlation between surface tension of the adhesive and the mechanical strength of the bonded substrates [69,74,75,76]. Fluorinated waterborne PU (FWPU) adhesive is such an example in which different amounts of amino- modified SiO_2_ nanoparticles and 2,2,3,4,4,4-hexafluorobutyl methacrylate (HFBMA) influenced the decrease of surface tension and the drop of the contact angle of the adhesive layer [69]. Even if in this case, the increase of contact angle was a proof of water-resistant formulations, while the decrease of surface tension indicated the increase of mechanical properties (depending on the concentration of silica nanoparticles), this behavior is not universally accepted. In our opinion, the performance of adhesives has to be correlated with a great deal of other electrostatic, chemical, diffusion, and mechanical factors not fully elucidated in terms of adhesion theory until now [69,77]. Thus, in such formulations an optimum concentration of the nanoparticles must be found to obtain best adhesion properties.

### 2.2. New Trends in Polymer Waste Management for Adhesive Formulations

Currently, most of the structural adhesives are synthetic polymers obtained from non-renewable fossil fuel resources. Moreover, the exploitation of fossil fuel is associated with pollution and the continuous increase and release of “greenhouse gases” into the environment. Compared to classic fasteners, structural adhesives are preferred by the automotive and aerospace industry considering the advantages that they bring in terms of mechanical and technological requirements [78], as well as lighter weight. Unfortunately, difficulties in the recycling methodology can emerge, due to the small portion of adhesive in the final components, and the fact that using polymers for adhesive bonds produces complex material combinations that are difficult to separate and, hence, recycle. ABJs are designed for high adhesion performance and their destruction and removal from the substrates are nearly impossible [79,80], which poses some difficulties in maintenance or recycling of bonded materials. It should also be stressed that there is a trend in what regards the use of thermoplastic-based adhesive formulations, since they can be recycled or reused by re-melting, contrary to thermosets, which are typically used for structural parts and are currently not being recycled. In this sense, providing structural properties to thermoplastic-based adhesive formulations becomes the challenge.

**Figure 1:**
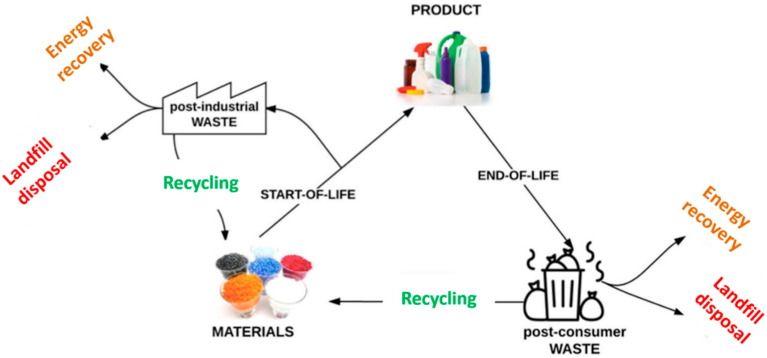
Lifecycle and routes for energy recovery, landfill disposal and recycling of plastic materials (Adapted from [82]. Reprinted from Waste Management, Vol 69, Ragaert et al., Mechanical and chemical recycling of solid plastic waste, 24–58. Copyright (2017), with permission from Elsevier).

In 2018, global plastics production almost reached 360 million tonnes (metric tons), while in Europe it almost reached 62 million tonnes [81]. The resulted plastic waste is treated differently using various strategies such as incineration, landfill disposal, and recycling (Figure 1) [71]. Landfill disposal has been banned in several countries. In the European Union, the incineration of plastic waste for energy recovery represents almost 40% of all strategies, which produces, annually, millions of tonnes of CO_2_ emissions [82]. Considering these aspects, some new trends in adhesive formulations are currently related to plastic and polymers waste recycling policies in order to ensure the circular economy “closing-loop” principle [80].

According to the document A European Strategy for Plastics in a Circular Economy released by the European Commission in 2018, a recycling rate increase to 50% of the total plastic waste must be reached by 2030 [83]. Based on these recycling policies, the tendency in structural adhesives is to apply chemical or mechanical recycling processes to plastic/polymer waste to valorize them to raw materials for new formulations.

Poly(ethylene terephthalate) (PET) is one of the most abundant polymers used for packaging or beverage industry. Until now, the continuous manufacturing of PET products led to the accumulations of large amounts of plastic wastes (51 wt% from the total amount of plastic wastes) [84]. PET can be recycled at four different levels: (i) primary recycling, involving the recycling (reutilization) of industrial scrap or waste in the form of raw materials to obtain the initial product quality; (ii) secondary recycling or physical recycling, involving contaminant removal, drying and melting processes of post-consumed PET products; (iii) tertiary recycling or chemical recycling, involving the depolymerization of PET chain into monomer units or larger oligomeric chains; and (iv) quaternary recycling involves the incineration of PET waste to produce energy [85]. In this review, we will focus on the tertiary and secondary recycling processes for synthesis of new raw materials for adhesive formulations and their performance in different binding systems.

#### 2.2.1. Chemical Recycling of Polymers for Adhesive Formulations

**Figure 2:**
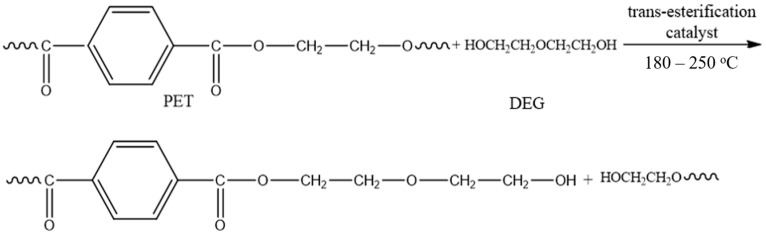
PET glycolysis reaction using diethylene glycol in the presence of trans-esterification catalyst (adapted from [92]).

PET depolymerization can be performed by employing solvolysis (aminolysis, ammonolysis, hydrolysis, glycolysis, and methanolysis) or pyrolysis processes, each with its own advantages and disadvantages [86,87,88,89,90]. Among these, PET glycolysis process is the most suitable for synthesis of raw materials for polyurethane or epoxy adhesives, as well as plasticizers for adhesives leading to manufacturing products with reduced cost [84,91,92]. The glycolysis process involves the utilization of small amounts of ethylene glycol or longer chain glycols in the presence of transesterification catalysts at temperatures ranging from 180 to 220 °C for 0.5 to 8 h, under conventional heating conditions or under microwave irradiation (Figure 2) [93,94,95,96,97].

The final glycolyzed products can be a mixture of aliphatic glycol, 1,4-butanediol, oligo- or polyether polyol and oligo- or polyester polyol with hydroxyl end-groups with relatively low molecular weight depending on reaction conditions [98,99]. Although the resulted oligoesters can be reacted with aliphatic acids to obtain polyester polyols with decreased hydroxyl number, the purpose is to use the final products as such in order to obtain adhesives with reduced manufacturing costs.

For instance, the depolymerization of PET in the presence of diethylene glycol (DEG), using aPET:DEG = 2:1 weight ratio, under microwave irradiation at 220 °C and using zinc acetate as trans-esterification catalyst led to the formation of oligoesters, with relatively reduced molecular weights of 1503 g/mol (78.55%), 507 g/mol (17.4%), and 209 g/mol (4.05%), that were further reacted with MDI in a typical PU adhesive formulation. In this case, the PU adhesive was used for wood joints and registered a medium shear strength of 6.7 MPa [92].

In some new proposed formulations of one-component diglycidyl ether of bis-phenol A (DGEBA) based epoxy resin, a derivate of terephthalic dihydrazide (TDH) obtained from PET bottle waste was used as curing agent. The idea was to reduce the curing time of the epoxy adhesive in structural bonding of aluminum to aluminum (Al/Al) [100]. This approach was well succeeded in the replacement of dicyandiamide, since it led to comparable properties of commercial epoxy formulation of the structural adhesive in which dicyandiamide is used instead of TDH.

Adhesive formulations, namely poly(vinyl acetate) (PVAc) adhesives, can be improved in terms of plasticizing components, since recent studies revealed that phthalate based plasticizers can be replaced with more environmentally friendly materials for this type of adhesives [84]. Polyesters were obtained by reacting liquefied wood or glycolyzed products resulted from PET depolymerization (in the presence of polyethylene glycol) with adipic acid (assigned as PE-LW and PS-PET, respectively). Following this reaction, the aqueous solution of PVAc was modified with PS-PET plasticizer (0–25 wt.%) for wood to wood joints. The performance of PVAc adhesives was found to reach commercial mechanical properties, even when smaller quantities of plasticizers are used (8.8 wt.% PS-PET and 20 wt.% PE-LW), compared to commercial phthalates, in which the amount of plasticizer goes up to 50 wt.% [84].

#### 2.2.2. Mechanical Recycling of Polymers for Adhesive Formulations

Waste polymers or polymer matrix composite materials with complex structures that are difficult/impossible to be recycled by tertiary or quaternary recycling techniques can be physically recycled by mechanical size reduction processes and used to improve mechanical properties of adhesive materials [82]. One example is the incorporation of grounded elastomer wastes in adhesive formulations aimed at increasing the flexibility of the adhesive material. In particular, the flexibility of PU adhesives can be tailored by the incorporation of neoprene rubber solutions (using toluene as solvent) [92]. According to our knowledge, this type of formulation in which certain types of rubber solutions are added, is more appropriate for rubber-rubber or rubber-leather joints. It can also be employed in wood-wood joints when the requirement is to increase the shear stress and flexibility of the ABJ. Adding rubber waste powder in solvent-free adhesive compositions can also constitute an interesting method to improve mechanical properties of the final bond.

Thus, the use of waste elastomers was investigated in the case of epoxy structural adhesive formulations in which the shear strength of epoxy-based recipe of DGEBA cured with triethylenetetramine (TETA) was improved by 64% for steel-epoxy carbon fiber composite joint, by just using 10 wt.% of recycled tire waste powder [101]. This type of formulations not only consumes and valorizes rubber wastes, but also decreases the manufacturing costs of the final adhesive, while improving the shear strength of the final ABJ.

It can be concluded that chemical and mechanical recycling approaches are tremendously useful methods that contribute to the reduction of plastic/polymer wastes, opening at the same time new directions for future commercial adhesive products with good performance and reduced manufacturing costs.

### 2.3. Microencapsulation of Isocyanate Species for Eco-Innovative Adhesives Formulations

High quality, strong, and long-lasting adhesives used in the footwear, construction, automotive, and aerospace industries, such as those based on PU, polyurea (PUa), and polychloroprene (PCP), typically include highly reactive isocyanate species in their formulation, as cross-linking agents, to provide the required high strength ABJs. However, isocyanates’ high toxicity is a primary concern when applying a two-component formulation (2K) PU or PCP adhesive, and the current safety regulations that limit its use in the industry must be taken into account [102,103].

The ongoing need for minimizing the hazards associated with the various components, as well as product degradation is the driving force for the exploration of microencapsulation in the adhesives field. Indeed, microencapsulation (MCs) is a promising solution to minimize the risks related to the handling and storage of hazardous ingredients included in adhesive formulations. Dominating this field is an opportunity to innovation in order to address the challenge posed by current and future safety regulations.

The first work, reported in the literature, on the microencapsulation of isocyanate compounds in the liquid state dates from 2008, with the encapsulation of isophorone diisocyanate (IPDI) targeting an application for self-healing polymers [104]. Recently, this type of MCs has been optimized and tailored for other applications, regarding adhesives formulations. PUa/PU shell MCs containing high loadings of IPDI in the core were developed to enable the production of mono-component, eco-friendly, and safer adhesive formulations for the footwear industry [105]. MCs containing not only IPDI, but also commercial isocyanate oligomeric and prepolymeric species, have been successfully developed [105,106,107] for this particular application, but also for other applications, such as self-healing in epoxy resin matrices [108,109].

The developed MCs are envisaged to have the following characteristics: high core content, a long shelf-life, mechanical and chemical resistance, and be able to release all the encapsulated isocyanate at the moment of the ABJ´s preparation, triggered by mechanical and thermal stimuli.

The microencapsulation of isocyanate is typically achieved by an oil-in-water (O/W) micro-emulsion system combined with interfacial polymerization, which involves the addition of, at least, two reactants in a pair of immiscible liquids. One of the liquids is preferably an aqueous solution, forming the continuous phase (W phase) while the other, the dispersed phase, is composed by the isocyanate to encapsulate (O phase) in the presence of an emulsifier/surfactant. Both phases (water-based and oil-based solution) contain reactive species (OH groups and isocyanate (NCO) groups, respectively) which react together to form an initial thin PUa or PU polymeric shell. The polymerization is controlled mostly by diffusion, so the growth rate of the microcapsules (MCs) shell will decrease as the shell thickness increases [110].

The thermal and mechanical resistance of the MCs´ shell can be tailored by the addition of “latent” active hydrogen (H) sources that are not readily able to react with the isocyanate NCO groups. For instance, by addition of silanes (e.g., aminosilane and n-octyl triethoxysilane, n-OTES) in W phase, silanol Si-OH groups will be formed, which will react with NCO, forming urethane moieties. By condensation reactions (Si–O–Si linkages), additional improvement of the shell’s mechanical properties and hydrophobicity are expected [105,107].

Better encapsulation efficiency is achieved by using a more reactive isocyanate than the one to be encapsulated, which will act as shell forming material. For instance, a commercial type of oligomeric MDI with increased functionality, Ongronat^®^ 2500 (Kazincbarcika, Hungary), with higher reactivity than IPDI, was employed as shell forming material [105]. Four different active H sources were tested, namely 3-(2-aminoethylamino) propyltrimethoxysilane (APTMS), tetraethyl orthosilicate, diethylenetriamine (DETA), and 3-isocyanatopropyltriethoxysilane (IPES), aiming at achieving a high encapsulation yield. The incorporation of a multifunctional isocyanate silane in the O phase, as “latent” active H source, led to the formation of impermeable PUa/PU-silica hybrid shell MCs (I MCs) with more than 60 wt.% of pure encapsulated IPDI [105].

The resulting MCs were incorporated in adhesive formulations and ABJs were prepared and compared with formulation without encapsulation of IPDI. The ones with encapsulated IPDI were found to exhibit the same peeling strength as the ones with non-encapsulated IPDI, which reveals the effective release and cross-linking ability of the encapsulated IPDI, in the form of a new generation of greener mono-component adhesives (Table 3).

MCs encapsulating IPDI and MDI pre-polymer via O/W emulsion with Arabic gum as a prediluted emulsifier, for adhesive formulations purpose, were also obtained via an innovative process, consisting of a microfluidic device [106]. The main advantage of this emulsification method is the ability to produce a narrow MCs size distribution. The MCs obtained via this new method were found to exhibit an IPDI encapsulation efficiency, as high as the traditional (batch) method, however, the yield of MCs obtention is not as high as by the batch process.

Moreover, it should be noted that the encapsulation of oligomeric, or pre-polymeric isocyanates by a PU/PUa shell is potentially preferred, instead of the microencapsulation of monomeric isocyanates, since it brings higher reactivity in what regards the formation of a 3D polymeric structure in the ABJs, or in self-healing applications. However, the encapsulation of these species, including the commercially available ones, is scarcely found in the literature and frequently involves synthesis issues due to high viscosities and difficulty to control the high reactivities, unless an organic solvent is added to the isocyanate solution (O phase) [107].

**Figure 3:**
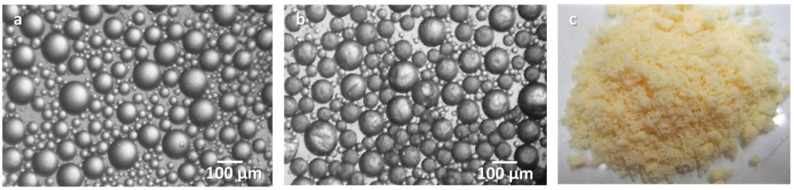
MCs morphology evolution during synthesis: (**a**) microemulsion before addition of the active H source, (**b**) shell formation after addition of the active H source, (**c**) free flowing powder consisting of the MCs after synthesis (scale 1:1). [107] Reprinted by permission from Springer Nature Customer Service Centre GmbH: Springer, Journal of Materials Science, “The role played by different active hydrogen sources in the microencapsulation of a commercial oligomeric diisocyanate”, by Loureiro, et al. (2020).

The microencapsulation of a highly reactive, commercial oligomeric MDI, Ongronat^®^ 2500, a medium viscosity liquid, solvent-free, with an increased functionality of 31 wt.% NCO, was recently reported [107], to be used as a crosslinker in PU-based adhesive formulations applications. In this work, the isocyanate species were encapsulated by a PU/PUa and a PU/PUa-silica hybrid shell, using a one-pot and straightforward approach, consisting of an O/W microemulsion system combined with an in situ polymerization at the interface of the O/W phases. The morphology of the MCs was studied along the synthesis, and a free-flowing powder was achieved at the end (Figure 3), ready to be incorporated into adhesive formulations.

The role played by a variety of amine based active H sources, namely ethylenediamine (EDA), APTMS and branched poly(ethyleneimine) (PEI), and the silane n-OTES, that simultaneously acts as a “latent” active H source and a hydrophobic agent was studied in terms of encapsulation efficiency, shelf-life, shell´s thermal stability and chemical structure for new potential adhesive formulations. The best overall performance was achieved for the combination of the high NH (amino) functionality PEI and the silane n-OTES, as active H sources (nOTES_PEI_MCs) with a loss of only 34% of the encapsulated isocyanate Ongronat^®^ 2500 during a period of seven months, in contrast with the 77.4% lost by the MCs synthesized without any additional active H source. They were found to be robust and provide an effective barrier against air’ moisture, which is critical for their future application as crosslinkers for eco-innovative 1K adhesive formulations [107].

More recently, MCs containing isocyanate made of a biodegradable shell have been reported, which further contributes to eco-innovative adhesives [109]. In this pioneering study, a biodegradable polymer, polycaprolactone (PCL), was used as shell material for the encapsulation of isocyanate species, which brings a further advantage, related with its thermal response in the temperature range of the adhesive joints manufacture. The obtained ABJs incorporating the MCs were found to exhibit the same peeling strength as the sample with non-encapsulated isocyanate, which reveals the effective IPDI release from the MCs core and its further reaction with the OH-prepolymer leading to a final crosslinked adhesive.

Thus, we highly believe that the encapsulation of high loadings of reactive isocyanate species enables their use as cross-linking agents in safer, eco-innovative, 1K and high-performing adhesive formulations, which can be further extended to other applications, such as self-healing approaches and smart materials.

### 2.4. Final Considerations on the State of the Art of Eco-Friendly Structural Adhesives

The strive for monomers derived from non-virgin petrochemical and bio-based raw materials, that are scalable, abundant and truly sustainable is a reality, and it is stimulating R&D activities at the global level and promoting the creation of new “green” businesses opportunities. Vegetable oils, wood derivates (sustainable biomass), polysaccharides, and recycled plastics are alternative feedstocks that are gaining importance in this field. However, mechanical properties of the adhesive performance are often compromised when moving away from conventional, well established petrochemical-derived plastics. Strategies, such as the incorporation of nanoparticles, or the microencapsulation of hazardous, but efficient cross-linkers are potential solutions to enable eco-friendly structural adhesives.

Finally, it should be stressed that a sustainability assessment combining the technical, economic/operational, environmental, and social dimensions should be implemented in what regards eco-friendly adhesives. However, such assessment, when applied to emergent materials and technologies, represents a significant challenge because there is a lack of proper indicators, lack of information, and contradictory information is common. This is the case of bio-derived adhesives, which might originate from nature-based materials harvested in a myriad of ways in several regions of the globe. Additionally, optimization of production systems is required to be technically and environmentally competitive.

Bio-adhesives derived from different available renewable biopolymers such as protein (soy) and lignin (Kraft and Organosolv), as well as tannin, were employed in a cradle-to-gate life cycle assessment or analysis (LCA) [111]. Other studies that compare bio- with petro-chemical adhesives, or potential greener processes, are found in [112,113,114,115,116]. A systematic literature review linking the sustainable assessment approach and biocomposite materials has been carried out [117]. The authors claim that life cycle sustainability assessment, integrating ecological, financial and social parameters, life cycle engineering, integrating the technical and functional aspects, and eco-design play a crucial role in encouraging life cycle thinking in decision-making for stakeholders, public authorities, and consumers, in these types of materials.

## 3. Surface Treatments to Enable Emergent Technologies

**Figure 4:**
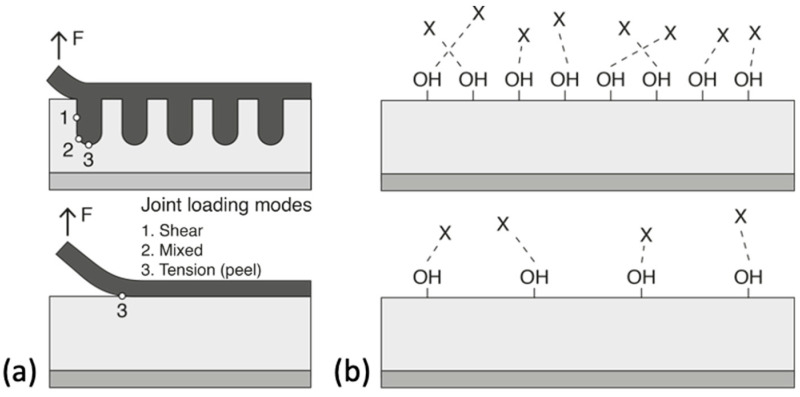
Schematic illustrations of the (**a**) mechanical advantage provided by surface treatments that increase the surface roughness and bonding area and (**b**) the effect of hydroxyl density on interfacial bonding with adhesive (represented by X). Reprinted with adjustments from [121] http://creativecommons.org/licenses/by/4.0/.

The creation of defined surface properties is the main goal to obtain reproducible bonding results—this is the decisive challenge for the effort in selecting a suitable pre-treatment [118]. Improving the adhesion between the substrate and the adhesive often requires modifying different surface properties that are known to influence adhesion. The modified properties can be either physical (mechanical), chemical, or, ideally, both. Physical modifications increase the surface roughness of the substrate to provide better wetting of the adhesive and a larger surface area. This contributes on a macroscopic level, as mechanical forces between the substrate and the adhesive also promotes adhesion. These types of interactions are introduced by changes in the surface morphology. According to the mechanical theory, adhesion occurs as a result of the adhesive penetrating into cavities, voids, or pores on the surface [119]. This theory is supported by experimental results showing an increase in joint strengths after mechanical roughening of the surface using grit blasting or mechanical abrasion [120]. Further, even greater strengths are achieved by the presence of an open porous structure formed by anodizing aluminium in acid electrolytes that leads to micro- and nano- surface roughness. This phenomenon is referred to as “mechanical interlocking”. The interface is then seen as a composite layer that enables better stress distribution and arrests the propagation of cracks during mechanical stress, see Figure 4a. Too much roughness, however, also has its disadvantages as it can lead to incomplete initial wetting of the surface and to the creation of voids that can act as failure initiation points due to stress concentrations [119]. Chemical modification of the surface improves its ability of the surface to form chemical bonds with the polymeric adhesive providing functional groups (e.g., silanes, hydroxides) that are compatible with the chosen adhesive and/or by increasing the density of these functional groups (Figure 4b). Acid etching, anodizing, and the use of coupling agents are the most commonly used methods to functionalize metal substrate surfaces. In practice, chemical and mechanical contributions are interrelated. After all, an increase in the surface roughness also leads to an increase in the surface area available for molecular and atomic interactions.

There is a variety of adhesion theories, such as mechanical interlocking, electrostatic adhesion, wettability, surface energy and thermodynamic adhesion, diffusion, weak boundary layer, and acid-base and covalent bonding theory, which can be applied for numerous substrate-adherent combinations, including natural materials, such as wood substrates.

Various processes are available to introduce both types of modifications, physical and chemical, which must be selected depending on the substrate, the adhesive, and the application requirements.

Depending on the given conditions, three main groups of surface treatment must be considered:

- Surface preparation [122], including cleaning [122] (removing dust, rust, contaminants like oils and grease), and geometrical adjustment (deburring);
- Surface pre-treatment, which can be physical-mechanical, physical, and chemical;
- Surface post-treatment, which contains the application of primers and the climatization.

This section addresses these three main groups of surface treatment, including process recommendations often derived from practical experience, and gives a critical insight about related REACH regulations. It concludes with a wrap-up where final considerations are given about this extensive topic.

### 3.1. REACH in Surface Pre-Treatments

The main drive for the exploration and development of new-generation surface pre-treatments comes from REACH regulations on the use of hexavalent chromium-containing products (Regulation (EC) No. 1907/2006) and volatile organic solvents (VOC Solvents Directive 1999/13/EC)

In 2006 REACH published new regulations for the future ban on hexavalent chromium (Cr(VI)). Chromates are marked as SVHC due to their carcinogenic and toxic properties. Any exposure during manufacturing or upon improper disposal of chemicals presents a very high health and environmental risks. In ABJ this mostly concerns the production stage when the parts are treated in preparation for bonding. For many years, Cr(VI) has been one of the most effective products for surface modification of metals and active corrosion-protecting pigments within adhesives and coatings. Chromates enabled to manufacture adhesively bonded aluminum sheets that are suitable for load-bearing structural aircraft components with a guaranteed lifetime of more than 30 years.

Hence, replacing chromates is not an easy task, not only due to the versatile nature of its benefits, but also since it affects companies from chemical suppliers to pre-treatment plants and downstream companies in different industries. The sunset date for the use of Cr(VI) was September 2017 and pre-treatments in many industries (e.g., automotive) do not rely on this substance anymore. However, an extension has been granted for some critical applications and it is still unclear for how many years further authorization will be granted, despite all the scrutiny around this substance.

### 3.2. Surface Preparation

#### 3.2.1. Cleaning

Cleaning is one of the most important steps when joining substrates together by adhesive bonding. If possible, oil or fat-containing residues should be removed with the aid of aqueous materials. The use of non-ionic cleaning agents leads to good results. Commercially available basic cleaning agents are particularly suitable for metals, as they not only remove the hydrogen carbonates; but stronger types can also remove metallic soaps and salts.

Some commercial mixtures are applied hot, while others use either anode or cathode currents. Regardless of the detergent used, the components must always be thoroughly rinsed. This supposedly minor aspect has turned out to be important in our daily practice, as surfactants on the surface simulate good wettability, but can act as a separating layer, or worse, as cross-linking inhibitors (e.g., sulfone-containing cleaners that prevent the curing of silicones) [123].

If the use of solvents is necessary to remove identification marks or paints, propan-2-ol should be used whenever possible. If this is not possible, a ketone shall be used alternatively. Solvents can cause severe damage to some thermoplastic materials either by dissolution or by initiating stress cracking. Plastics based on polycarbonate, polymethyl methacrylate, and acrylonitrile-butadiene-styrene are particularly susceptible to stress cracking.

Ultrasonic cleaning may also prove acceptable for the pre-treatment of smaller components.

For the pre-treatment of titanium and its alloys, very effective steam baths are recommended. However, these may only be used in closed systems.

It should not be forgotten that some industrial processes can and do have harmful effects on surfaces, both during and after their pre-treatment. With the use of equipment is often accompanied by the release of harmful dust, smoke clouds and vapors into the surrounding air. Oil vapors, mold release sprays and the air in electroplating plants are particularly harmful. For this reason, surface pre-treatment (and bonding) should be carried out in separate rooms where such contamination can be avoided.

#### 3.2.2. Cleaning with Laser

The laser process removes dirt and top layers without residue only by means of light. No chemicals or other media (as in pellet or sand blasting) are required. The particles, which are produced in small quantities, can be extracted immediately after laser ablation, and disposed.

Laser ablation occurs when a layer of a material or a material which is deposited on a given surface is removed with the aid of a laser beam. Contaminants can by removed selectively with little impact on the substrate. Given a sufficiently large ablation threshold difference between the materials, it is possible to select one material to be removed (the one with the lower ablation threshold), leaving the other material untouched. Uniform and reproducible cleaning results are obtained [124,125].

### 3.3. Surface Pre-Treatment: Physical (Mechanical) Treatment

Roughening the surfaces of both, metals and plastics, either by grinding or by blasting, is almost always suitable for improving the performance of the bonded joint ultimately achieved. Sometimes grinding or blasting is required to smooth a rough, uneven surface, such as that of castings, or to remove corrosion or other types of contamination [122].

#### 3.3.1. Grinding

Grinding can be carried out either wet or dry using either a water-resistant coated paper (45 µm to 106 µm grain size) or a three-dimensional non-woven abrasive.

The following sequence of operations is recommended:

(a) In a direction convenient to the performer, sand straight across the surface until the entire surface is lightly and evenly roughened;
(b) In the same way, sand at right angles until all traces (of (a)) are removed;
(c) Using a circular motion (with a diameter of: <100 mm), grind until all traces of the previous step (see (b)) have been removed and the surface appears uniform;
(d) The grinding residues shall be removed. If dry sanding has been carried out, use a vacuum extractor, if applicable. Otherwise, clean, oil-free and dry air must be blown into a suitably ventilated environment. If wet sanding has been used, wipe the solvent off the object with a clean, lint-free piece of cloth and let it dry;
(e) Followed by either gluing or another surface modification procedure.

If parts are to be bonded, they shall be dry and bonded as soon as practicable. Drying may be accelerated by means of a clean, oil-free, hot air-drying stream at a temperature not exceeding 60 °C.

#### 3.3.2. Blasting

**Figure 5:**
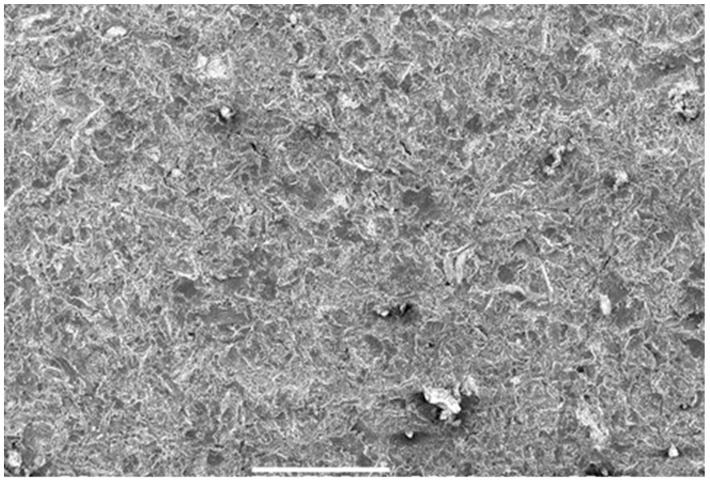
SEM picture after blasting an unalloyed steel (S355) with corundum of average grain size F022 (710–1000 µm). Scale bar = 500 μm. (TU Braunschweig).

Dry blasting is mainly used for metallic components. However, less aggressive methods may prove effective on more robust plastics and when used with care, to avoid excessive erosion. Legally-protected processes are available for this purpose. These include the use of such special blasting agents as granulated carbon dioxide or shredded nutshells. In general, however, the metallic components are pre-treated by dry blasting with an abrasive of various grain size until the surface appears uniform. In steel construction, the blasting process for unalloyed steels has the task of achieving a surface quality Sa 2^1/2^ according to EN ISO 8501-1, by removing the layers of scale and rust [126]. In some cases, residues of the blasting material can be found on the surface after blowing off (Figure 5).

Although the process is complex in a construction site environment, it is still preferred [127,128]. For parts made of aluminum, copper, stainless steel, or titanium no iron or steel-based abrasives may be used. Typical abrasives are corundum (Al_2_O_3_), glass, ceramic, or stainless steel (CrNi) beads.

Pre-treatments with CO_2_ as a blasting material results in thermal, mechanical, and expansive effects [129,130,131], where the ice or snow will cool the surface and make it brittle. The mechanical effect comes from the kinetic energy of the CO_2_ particles which can reach supersonic speed and the expansive effect comes from the sublimation.

The effect from CO_2_ blasting is a cleaning and re-oxidation of the surface and it has the potential to increasing the surface roughness. A strain hardening can also sometimes be observed but the surface energy will generally not increase. A combination with silane primer is known as the Cryosil^®^ process [132,133].

Silane-coated corundum is used in the so-called SACO^®^ or Rocatec^TM^ process. The silane layer is removed in a triboplasmic process when the corundum grain hits the substrate, remaining on the substrate surface. The use of such an in situ coating has been considered for conventional overpressure and low-pressure blasting [134]. Since the effect of the in situ deposited adhesion promoter layer often becomes apparent only after ageing exposure, test specimens have been additionally subjected to storage in a high-temperature and moisture-saturated atmosphere.

Lap shear tests on, e.g., aluminum-aluminum components bonded with a two-component epoxy resin adhesive showed a typical post-curing effect caused by the damp-warm aging and a higher joint strength of the SACO-blasted samples due to the surface preservation. Furthermore, the samples pre-treated by means of low-pressure blasting achieve identical strengths as the samples produced in the conventional overpressure blasting process. Fusion bonded components of carbon fiber reinforced thermoplastic tapes (CFR-TP) with blasted steel surfaces having been analyzed [135]. The results obtained by SACO^®^ blasting show a higher peeling resistance and an improved failure mode than those obtained by blasting with corundum (either at low-pressure or overpressure) or plasma pre-treatment.

The Rocatec^TM^ process, on the other hand, has been used for medical applications as a standard procedure, especially in the pre-treatment of titanium [136,137] in dentistry and for implants.

Wet blasting performed at an angle of less than 90° to the surface, using a sieve of <20 µm suspended either in water or in steam, may be particularly suitable for small metallic parts. The legally-protected systems usually contain water-soluble additives. To avoid further contamination of the surface, the advice of the manufacturer should, therefore, be sought.

#### 3.3.3. Low-Pressure Blasting

**Figure 6:**
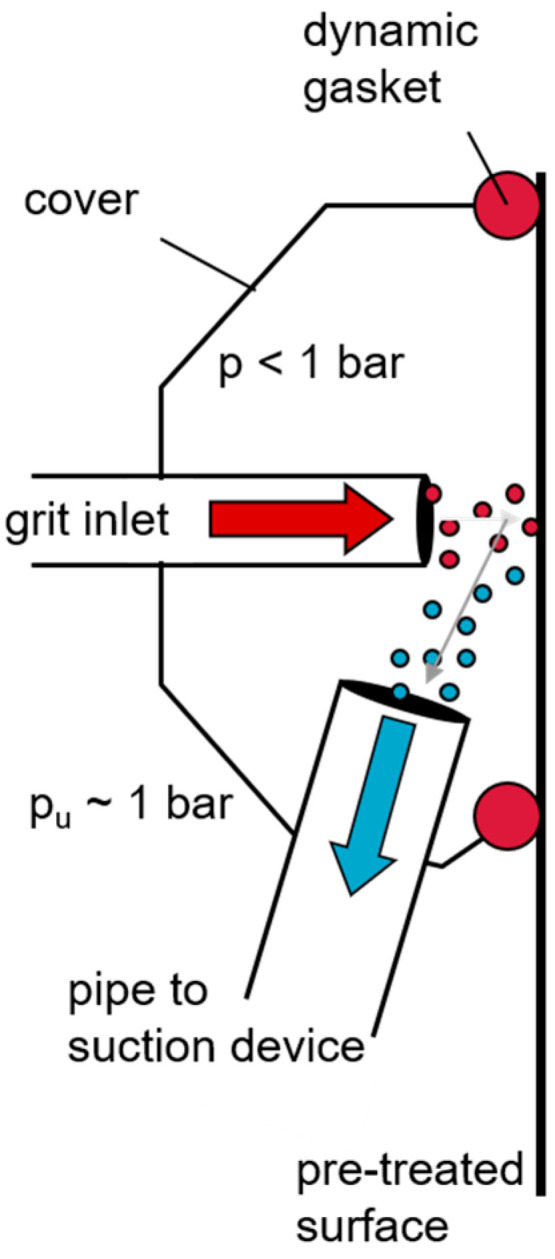
Schematic design of a low-pressure blasting device. Adapted from [138].

As with conventional overpressure blasting, particles are accelerated by the kinetic energy of an air stream onto the surface of the substrate. In contrast to the conventional blasting process, however, vacuum blasting, the gas flow in generated through a negative pressure. The machining process is locally enclosed using a blasting hood (Figure 6). This offers the essential advantage that the entire structure forms a closed system [132,136,137,138,139].

Low-pressure or vacuum blasting is an environmentally friendly method that can be used as a pre-treatment for composites (fiber-reinforced plastics) as well as metals. In our own investigations it has proven to be a good alternative for small series production in aviation and automotive industry.

#### 3.3.4. Peel Ply

Peel plies are applied to composites during their manufacture [140]. They protect the composite surface from environmental degradation and bruising during transport and storage. Additionally, if further work, such as adhesive bonding, is required, the removal of the peel ply produces a clean surface with the required roughness. Ideally, peel ply can replace abrasive work phases, such as sanding or grit blasting. However, it should also be stressed that peel plies have also been reported to contaminate the surface [141], so that a careful evaluation should be made on a case by case basis.

### 3.4. Surface Pre-Treatment: Other Physical Treatments

Processes which modify surfaces without the use of mechanical grinding or liquid-based chemical are part of this chapter. These processes are essentially designed to enable advantageous chemical modification of the surfaces of, especially, plastics by physically-initiated oxidation processes. Some of these processes also introduce other chemical elements during the modification of the treated surface. With the help of some of these processes, impurities can also be removed to a certain extent.

All processes require optimum conditions to be ensured, and it is proposed to develop suitable process parameters to get the best result.

#### 3.4.1. Surface Modification by Oxidizing Flame

Surface modification by an oxidizing gas flame is a relatively simple, rapid, effective, and economical method of improving the surfaces of a wide range of plastics. This process is particularly suitable for the pre-treatment of complex, three-dimensional components.

It should be noted that careful automatic control of the process parameters is important and that the parameters must be selected in relation to the polymer to be treated and the component design.

In order to achieve uniform results, special attention must be paid to

(a) The gaseous fuel used;
(b) The residual oxygen content of the flame;
(c) The distance between flame and surface;
(d) The speed with which the flame moves over the surface;
(e) The standardization of the time sequence before gluing.

#### 3.4.2. Atmospheric Pressure Plasma

There are different types of atmospheric pressure plasma like plasma torches, dielectric barrier discharge, and radio frequency capacity discharge plasmas for surface treatment before bonding. They can be used with air or different treatment gases alone or in combination with precursors for plasma polymerization [142,143,144,145,146].

Plasma treatment results in a gentle cleaning, forming functional groups and, in some cases, a structural change on the nanometer level. The increase in adhesion is related to the potential to form functional groups like CH_2_OH, C–O, C=O, and O–C–O and a reduction of C=C and CH_2_ bonds. They are formed during plasma treatment of shortly after with ambient air. In addition, and according to our own results, in the absence of oxygen, e.g., under nitrogen/hydrogen atmosphere, the plasma leads to a strong improvement of the joint by nitrogen-functionalization of the surface [146]. In general, the increase in polar groups improves the surface energy and leads to a good wettability. However, the increase in functional groups on the surface will deviate with time for an open and not-bonded surface.

#### 3.4.3. Corona Discharge

Corona pre-treatment regards a high-voltage discharge between an electrode and a counter-electrode. The electrical discharge ionizes the air present in the electrode gap. When a plastic film or flat component is passed through, the reactive species created by the discharge interact with the polymer surface and activate it. As in plasma activation, oxygen-containing groups are introduced into the upper molecular layers, which increase the polar fraction of the surface energy [118,142,146,147].

A distinction must be made:

Direct corona-discharge between electrode and grounded counter-electrode, being the treatment material guided through the gap; and

Indirect corona-discharge between two ceramic electrodes, where the resulting “current filaments” are directed by a laminar air flow onto the surface to be treated.

#### 3.4.4. Low-Pressure Plasma Discharge

Low-pressure plasma discharge processes can be considered more adaptable than flame oxidation processes. Complex shapes are generally not a problem and surface modification can be optimized by using different gas combinations in the discharge chamber. However, the attractiveness of this process is diminished both by the high cost of the equipment required and by the fact that plasma chambers require a batch-based process, unlike gas flame and corona discharge processes, which can be carried out continuously. The effect of low-pressure plasma is similar to atmospheric plasma [144,148,149,150].

A recent study shows that oxidized short polymer fragments generated by oxygen low-pressure plasma can be washed off by solvents, thus restoring the initial, as-received chemical surface. However, this did not reduce the adhesive strength of the obtained ABJs. For this reason surface functionalization as the dominating factor for a high quality ABJ has to be re-evaluated [151].

#### 3.4.5. Laser

Laser beams have also been used for the pre-treatment of surfaces of both, metals, and plastics.

There are many different types of lasers for pre-treatment before bonding, working in the infrared (IR) or ultraviolet (UV) range of the electromagnetic spectrum.

IR lasers show a cleaning effect from thermal removal of contaminants. For metals this results in the lowering in carbon from organic contaminants or films and removal of inorganic particles from cutting and forming [152,153,154,155,156,157,158,159,160,161,162,163].

**Figure 7:**
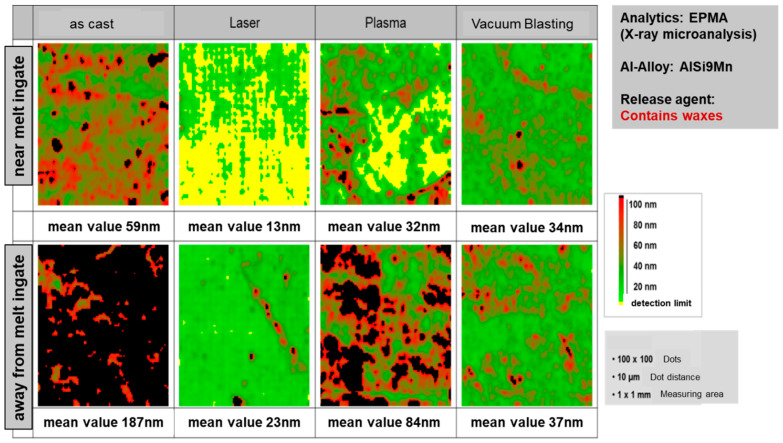
EPMA (X-ray microanalysis; carbon content) of the aluminum die-cast surface after various pre-treatments. Adapted from [164].

The distribution of carbon residues on a substrate surface, consisting of a casting aluminium alloy EN AC-AlSi9Mn, after different pre-treatments (laser, plasma, vacuum blasting) is shown in Figure 7. Laser pre-treatment is the most effective to remove waxes from the surface of a die-cast aluminum surface.

**Figure 8:**
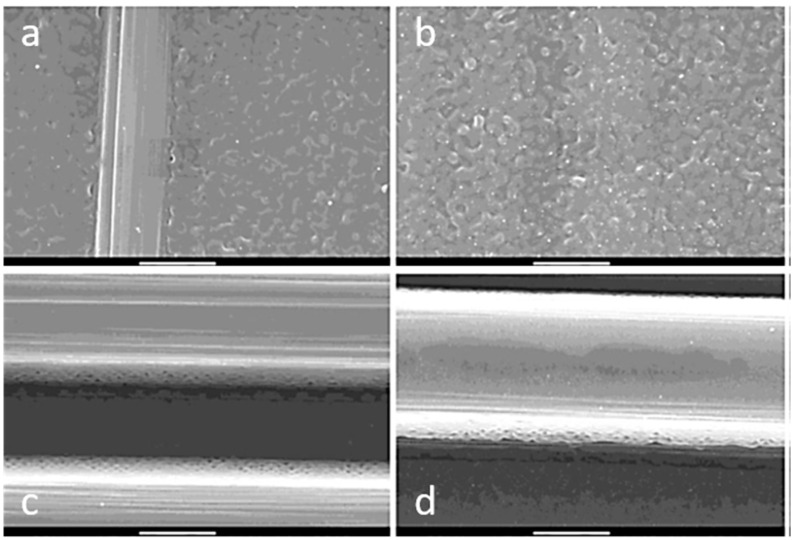
SEM pictures of laser-treated CFRP surfaces. (**a**): two pulses, 600 J/cm^3^; (**b**): two pulses, 800 J/cm^3^; (**c**): 16 pulses, 600 J/cm^3^; (**d**): 16 pulses, 800 J/cm^3^. Scale bar = 5 μm [157]. Reprinted from Phys. Procedia, Vol 41, Kreling et al. Analytical characterization of CFRP laser treated by excimer laser radiation, 282–290. Copyright (2013), with permission from Elsevier.

The laser energy is absorbed by the surface and can result in a re-melting and thermal oxidation of the surface. This will affect the surface structure and wetting, as well as the thickness, porosity of the oxide layer, bonding strength and, sometimes, the surface hardness. For CFRP, IR laser pre-treatment can be used for removal of the top resin layer, enabling the bonding between adhesive and fiber. However, the absorbed radiation might heat the fibers and, thus, degrade the matrix and may damage the integrity of the composite. Figure 8 exhibits specimens treated with 16 laser pulses, which show clearly-exposed fibers independent of the pulse energy. In the border area of the fibers, a small structure can be noticed which might hint to some local damage of the sizing.

UV lasers show a cleaning effect by photoablation process. This results essentially in the absence of a heat affected zone. They can be used for cleaning and structuring and to improve wettability. The natural oxide layer present at the surface of metallic substrates can be removed, as well as mold release agents present on CFRP substrates.

A comparison of mechanical and laser pre-treatment shows that the laser has the potential to substitute mechanical processes, with advantages at the level of environmental effects [165].

### 3.5. Surface Pre-Treatment: Chemical Treatment

For critical bonding applications the previously mentioned processes are often not sufficient to produce a long-lasting adhesive bond. Therefore, chemical and/or electrochemical treatments are often required. Although chemical surface treatments are a relatively simple procedure, in practice many competing reactions can occur and the slight variation in process conditions, such as concentration of additives, the bath temperature, and the ion content of the substrate metals, make this process more complex than physical pre-treatments. It is important to keep in mind that chemical treatments produce large amounts of chemical wastes, which is a major environmental issue. These treatments often employ strong oxidizing agents that require careful handling and subsequent disposal.

#### 3.5.1. Etching, Pickling

These treatments aim to remove the metal surface layers that are prone to degradation and instability due to microstructure abnormalities (including smaller grains, incorporated oxides, and modified chemical composition), as well as prepare a new and more stable oxide that is suitable for bonding. This can be performed in either acidic or alkaline solutions (hence the two different terms). When high copper containing alloys are alkaline etched, a characteristic smut is deposited, which must be removed by immersion in Cu-dissolving medium, typically a HNO_3_ solution.

#### 3.5.2. Conversion Coating

A special type of chemical modifications are conversion coatings. These coatings are formed by precipitation of certain metal oxides that are used to form a thin, but stable, layer on the surface of different metals. These layers improve the adhesion of subsequently applied organic layers, as well as providing a barrier to the environment, often releasing corrosion-inhibiting species. The most important new generation of conversion coatings are based on titanium and/or zirconium chemistry [166,167].

#### 3.5.3. Anodizing

**Figure 9:**
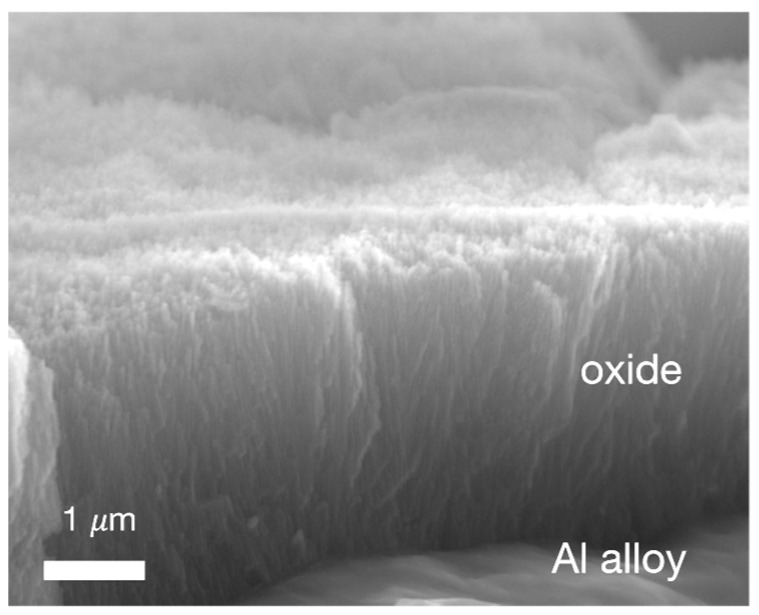
Example of a cross-section of an anodic aluminum oxide prepared by PSA anodizing (Vrije Universiteit Brussel).

Anodizing is one of the best commercial methods to create porous anodic oxide films on aluminum and titanium. These layers provide a nano-porous template (Figure 9) for the adhesive to infiltrate, thereby enhancing bonding and interface stability. Only few of the investigated greener anodizing processes have evolved to commercially mature industrial processes, and even less are suitable for primary bonding applications. Phosphoric acid anodizing (PAA) and phosphoric sulfuric acid anodizing (PSA) are the only processes that are adequate for adhesive bonding of primary structures in the aviation industry. Other anodizing processes, such as sulfuric acid anodizing (SAA) and tartaric sulfuric acid anodizing (TSA) can be used for bonding of non-primary structures and other fields (e.g., automotive). Anodizing is a complex process in which the nature of the electrolyte and the operating conditions are critical to the final chemical and morphological properties of the surface [121].

### 3.6. Surface Post-Treatment

Post-treatment is a term used to refer to all subsequent steps that take place after the pre-treatment. For bonded parts, these are mainly primer treatment with coupling agents.

#### 3.6.1. Coupling Agents

Adhesion promoters, or coupling agents, enhance adhesion between inorganic substrates and organic polymers (adhesives) by improving the stability of the interface through covalent chemical bonds. They are often chosen with dual functionality, containing organic end groups such as methoxy, ethoxy, or hydroxyl and an amino group that can interact with the adhesive, e.g., epoxides. Among coupling agents, silanes are used the most. The performance of coupling agents depends on the physical properties resulting from the method of application (thickness of the resulting film) as much as the chemistry (pH, stability of the solution) involved. As a result, coupling agents also improve the resistance to environmental degradation and disbanding [118,168].

#### 3.6.2. Primer

In regular manufacturing operations, there is a certain time delay between substrate pre-treatment and bonding (several hours up to several months). During this time, the freshly prepared surface is susceptible to damage, contaminations, and environmental degradation. To prevent this, the surface is covered by a thin layer of organic coatings, the primer. Primers are diluted polymeric coatings, usually matching the chemistry of the adhesive. The primer functions as a physical barrier between the pre-treated surface and its surrounding, possibly also containing coupling agents to promote adhesion and active corrosion-inhibiting species [122,169].

#### 3.6.3. Climatization

A distinction must be made between storage in the laboratory and delays occurring during industrial production. The former includes the performance evaluation of either the surface or the adhesive. If an assessment is required, storage shall be in an environment with a temperature of 23 ± 2 °C and a relative humidity of 50 ± 5%. The components shall be used within a period of 8 h, except for materials which are still subject to harmful oxidation processes, such as soft unalloyed steel. Such surfaces must be bonded as soon as possible after pre-treatment and always kept under specified conditions before bonding to avoid contamination [122].

Industrial production requires compliance with certain minimum quality standards. To this end, procedures must be established to ensure that the integrity of a pre-treated surface is not unacceptably damaged before joining. Particular attention shall be paid to possible damage caused by oxidation, condensation, and contamination, especially by mold release agents; these shall never be used in the same building. Ideally the parts should be glued immediately after pre-treatment and only exceptionally after a period of 4 h.

### 3.7. Final Considerations on the State of the Art of Surface Treatments

REACH regulations have stimulated the development of a diverse range of surface pre-treatments. Relevant processes for metals involve chemical, physical, or plasma treatment. For composites a chemical cleaning, gentle physical treatment, plasma, corona, flame, and peel ply are common pre-treatments. These processes offer a range of benefits, including cleaning from contaminants or corrosion products, to roughening the surface for an improved mechanical interlocking. In general, an improved wetting by the adhesives and a modified surface chemistry, often combined to achieve a higher level of performance, leads to stable ABJs, with enough resistance to aging.

Among the above described physical pre-treatments, plasma, corona, flame, and laser treatment are the ones that are mostly used automated, low space consuming and sustainable through little need of energy, water, and chemicals. Surface post-treatment with coupling agents or primers include chemistry that could be beneficial for ABJs but are from a sustainability point of view recommended mainly for applications with high environmental demands or when bonding is difficulty performed otherwise.

The results of LCA for adhesive bonding steps or even pre-treatments can vary a great deal depending on the methodology used or the system being investigated. It is, therefore, not possible to make general statements. D.F. Williams [170] made an LCA with the goal of comparing a conventional, wet-chemical process (oxalic acid etching) with an argon plasma treatment. A cradle-to-grave LCA was not appropriate in this case, but the differences between the processes were included in the LCA. This work consisted of a gate-to-gate assessment as it only considers what arrives at the factory and what leaves the factory. It was reported that when the efficiency of the chemical process is reduced to an application-like level, there is almost parity with the plasma process in regard to the climate change factor. In another study applied to footwear industry [171] three different polyurethane adhesive technologies and the specific primers were compared. A better management of the energy expended during the application step using renewable energy sources, improvement of equipment energy efficiency, and development of new formulations are potential possibilities to reduce impacts. In this context, it should also be pointed out that some adhesive manufacturers are evaluating their own products in terms of lifetime sustainability and developing primers with lower environment impact [172]. In a related field of bonding technology with aspects comparable to adhesion, i.e., the metallization of polymers, LCA was carried out [173], allowing to compare different surface treatments.

Chromic acid anodizing processes are currently being replaced in many industrial sectors. The whole life cycle of the process must be considered to ensure that the possible chosen solution for eliminating or lowering hexavalent chromium, Cr(VI), emissions would not drastically damage the overall process performance in other environmental areas. Relevant works [174] confirmed that the only way to efficiently deal with Cr(VI) compounds is to substitute the electrolyte used in the bath, since most of the Cr(VI) emissions are caused at secondary stages of the process.

As a result of such LCAs, many REACH-complaint surface pre-treatments have been adapted by varied sectors. However, it should be stressed that an analog replacement in the aerospace industry, with its high level of performance and safety, is a very challenging task. In addition, the time to test and qualify new systems for aviation is much longer compared to other industries. Therefore, some Cr(VI)-based substances are still used to assure the required level of performance in ABJs, while many other steps of the pre-treatment process already employ environmentally-friendlier alternatives. 

## 4. Disassembling Strategies for Maintenance and End-of-Life of Structural Adhesives

Today, many commercial adhesives are developed to reach the highest possible adhesive and cohesive strength and to last “forever.” As tempting as “forever” sounds, it is not desirable in many applications (e.g., products with high complexity or cost) since maintenance, repair works, or upgrades must be done from time to time [175].

Further, recycling at the end of the product’s life cycle must be considered. Environmental requirements, such as the EU End-of-Life Vehicle (ELV) Directive, demand that all cars must be recyclable at a minimum of 95% of the vehicle mass [176]. In all these cases, there is a need for adhesive technologies, which allow debonding of the surfaces. By debonding, or disassembly, we mean the breaking of the polymer chains into shorter sub-units and monomers, to aid maintenance, or recycling operations. Disassembly may be triggered at an appropriate time, either by a chemical or physical-assisted approaches.

**Figure 10:**
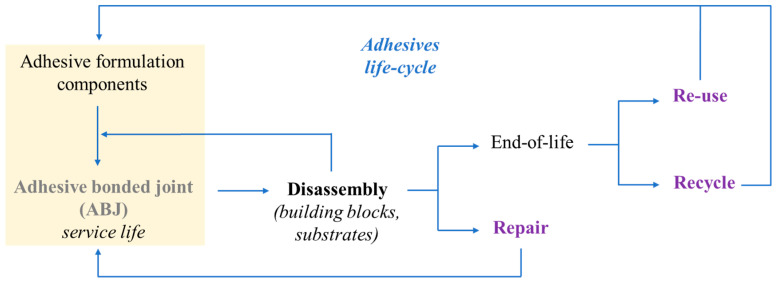
A life-cycle diagram, showing adhesive materials flows. Adapted from [177] “Overview of disbonding technologies for adhesive bonded joints”, A. Hutchinson, Y. Liu, et al. Journal of Adhesion, Vol 93, Pages No. 737–755, Copyright (2016), with permission from Taylor & Francis Ltd., http://www.tandfonline.com.

With this in mind, Figure 10 gives an overview of the materials flows within the life cycle of an adhesive-bonded joint, recalling that formulation components and adhesive structures should be designed with materials recovery in mind [177], to promote the reuse, repairing, and recycling of implicated materials.

There are many concepts and patents published [178,179,180,181,182,183,184,185,186,187,188,189,190,191] that deal with debonding surfaces, but only a few solutions are available on the market. All of them have their advantages, drawbacks, and limitations. In the following, we give an overview of the existing technologies concerning the debonding of structural adhesive bonds.

To bond structural joints with adhesives, the usage of epoxy glues and other thermosetting polymers are the gold standards. These adhesives are very stiff and durable and form a steady bond even at elevated temperatures.

### 4.1. Traditional Approaches: Using Force/Degradation of the Adhesive

Since thermosetting polymers form a permanent network during the curing process, the debonding of such adhesives is mainly based on mechanical and thermal destruction of this network. Therefore, the adhesives in the joints are thermally degraded, cut apart, or both are used in combination to debond the substrates. In other cases, solvents or acids are used to degrade the adhesives. Where complex geometries are bonded, knives, scrapers, and wires are used, resulting in high costs and adhesive residue still present after separation [177]. All those methods carry a high risk of damaging the substrate and are not applicable if the materials do not withstand the harsh conditions of disassembly [192].

### 4.2. Debonding on-Demand

A better solution to this process is to employ adhesive systems that allow a debonding on-demand. On-demand means the adhesion joint stays stable under working conditions, but in the event of maintenance, repair, upgrade, or recycling, an external trigger can weaken the bonding resulting in debonding without damaging the substrates. Ideally, there is a clean separation of the adhesive, and a substrate without remaining residues on one surface, so less residual adhesive must be removed.

The main strategy for debonding on-demand is based on the addition of functional additives to the adhesive system or on the modification of the adhesive itself. Incorporation of a debonding technology into an existing system or process can cause compatibility issues with the adhesive chemistry, or a negative impact on the rheology of the mixed materials. It must also be considered that the addition of additives can influence the mechanical properties of the polymer (binder), and the adhesive system might become too weak for structural bonding [177].

For structural adhesives debonding, on-demand is achieved by adding particles in the adhesive or the primer layer. The particles incorporated stay inactive while the bonded joint remains under working conditions. For initiating the debonding process, the particles can be activated by an external trigger. Different approaches are known, and some of them are already commercially available.

#### 4.2.1. Incorporation of Nanoparticles into the Primer Layer

One option to generate a debonding on-demand is the presence of selected nanoparticles in the primer layer. The primer layer improves the adhesion between the substrate and the adhesive by acting as a linker between them. Those particles should exhibit ferromagnetic, ferrimagnetic, superparamagnetic, or piezoelectric properties, so that, when subjected to an alternating electromagnetic field, they produce a large amount of localised heat. The heating happens very fast and melts or decomposes the adhesive material near the primer layer or the primer layer itself. This process causes a loss in strength so that the bonded joint can be easily debonded [179,193,194,195].

**Figure 11:**
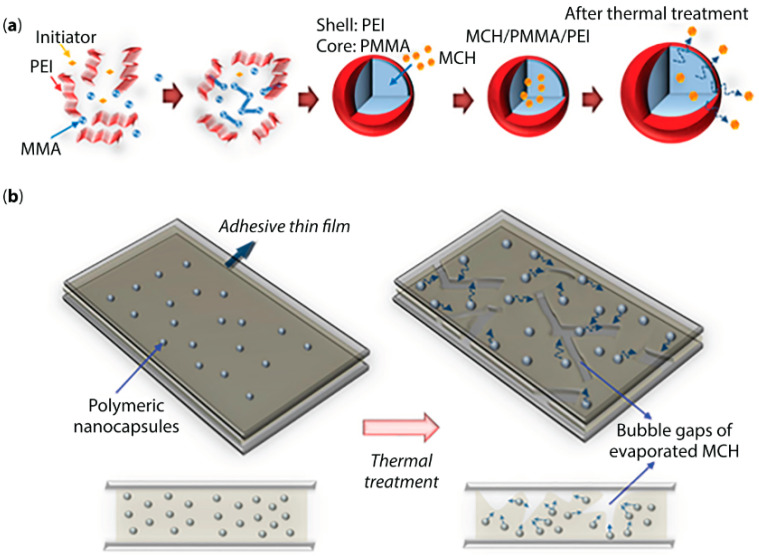
Debonding technique using polymeric nanocapsules with an adhesive thin film, which forms gas bubbles through thermal stimulus. (**a**) Synthesis concept of a polymeric nanocapsule containing internal vaporizing material, namely nanocapsules composed by hydrophobic methylcyclohexane (MCH)/poly(methyl methacrylate (PMMA) in the core and polyethyleneimine (PEI) in the shell. (**b**) Schematic representation of debonding mechanism through the formation of bubble gaps at the interface of a thin film adhesive, by heating the adhesive system [196]. Reprinted from Journal of Applied Polymer Science, Vol. 135, Issue 31, Lee et al. “Polymeric nanocapsules containing methylcyclohexane for improving thermally induced debonding of thin adhesive films”, Pages No. 10, Copyright (2020), with permission from John Wiley & Sons Inc.

Another way of weakening the bonding is to incorporate polymeric nanocapsules with low boiling point substances. When heated up, the substances evaporate and release gas into the interface of the bonded parts. The resulting gas bubbles weaken the bond, causing a more straightforward disassembly process (see Figure 11) [196]. After heat treatment, the adhesive bond still possesses about 50% of its initial strength. Therefore, additional force is still necessary to separate the bond, but much less than that required if no debonding process was applied.

#### 4.2.2. Incorporation of Microparticles into the Adhesive

##### Thermally-Expanding Particles

**Figure 12:**
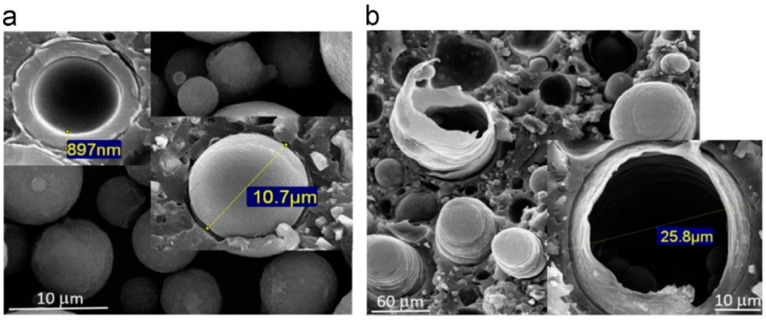
SEM image of TEPs before (**a**) and after expansion (**b**). [206] Reprinted from International Journal of Adhesion and Adhesives, Vol 54, Banea et al., “Mechanical and thermal characterization of a structural polyurethane adhesive modified with thermally expandable particles”, Pages No 191–199, Copyright (2014), with permission from Elsevier.

**Figure 13:**
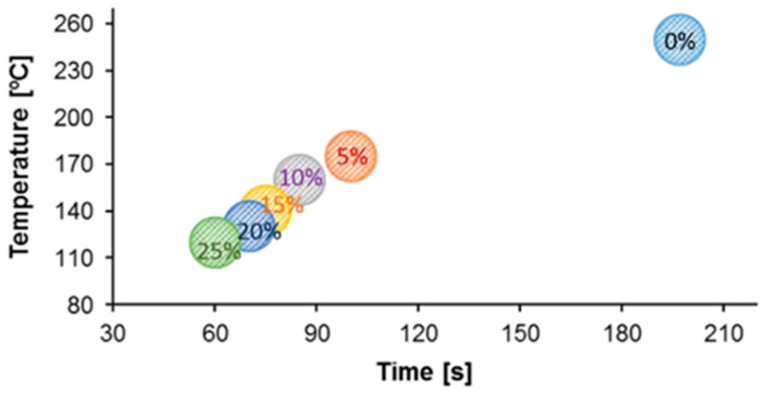
Results from debonding evaluation test (followed induction heating at 250 °C) employing a commercial adhesive used in the automotive industry (Betamate™2098). Temperature (°C) versus time for debonding. Percentage shows the amount (wt%) of TEP in the adhesive. The more filler that is added, the faster the bond fails and the lower the failing temperature [199]. Reprinted from International Journal of Adhesion and Adhesives, Vol 59, Banea et al. “Debonding on command of adhesive joints for the automotive industry”, Pages No 14–20, Copyright (2015), with permission from Elsevier.

Another strategy for debonding joints on-demand is by adding reactive fillers to the adhesive layer. Here the most know fillers are thermally expanding particles (TEPs). They were developed in the early 1970s by Dow Chemical Co. [188]. The particles consist of liquid hydrocarbon encapsulated by a thermoplastic shell. When heated, two transformations occur. First, the thermoplastic shell of the particles softens; second, the hydrocarbon evaporates (see Figure 12). The evaporation leads to an expansion, which can be up to 100 times in terms of volume. If cooled down again, the shell hardens, and the particles stay in their expanded state. This volume expansion leads to internal stress, cracks, and a reduction of density, which, combined, decrease the strength of the adhesive [197,198,199,200] (see Figure 13). This mechanism is well understood and found in many applications already [201,202,203,204,205]. Nevertheless, an introduction of higher amounts of TEPs to the adhesive leads to a weakening, as desired, of the ABJ. Further, the adhesive remains stuck to both substrates and has to be mechanically or thermally removed [200].

##### INDAR Inside

Another industrially-used approach is a technology called “INDAR inside”, which was developed by RESCOLL, and regards chemical expansion agents, such as p-toluenesulfonylhydrazide, azoicarbonamide, polycarboxylic acid, encapsulated by a wax matrix. These active particles are incorporated into an adhesive polymer base, and decompose under specific temperature, releasing gases, which diffuse from the bulk to the interfaces. Here the gases generate local stress leading to the debonding of the joint. Depending on the type of additives, the decomposition temperature can be varied, allowing tailoring of the debonding temperature. A further advantage is the adhesive failure of the bond, which means that one surface of the substrate is free from residual adhesive [181,207].

##### Expandable Graphite

**Figure 14:**
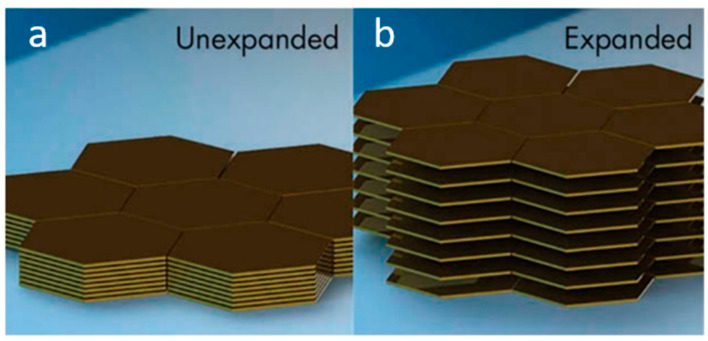
Graphite flakes unexpanded (**a**) and expanded (**b**). [177] Reprinted from “Overview of disbonding technologies for adhesive bonded joints”, A. Hutchinson, Y. Liu, et al. Journal of Adhesion, Vol 93, Pages No. 737–755, Copyright (2016), with permission from Taylor & Francis Ltd., http://www.tandfonline.com.

Some applications require relatively high curing temperatures, e.g., 180 °C in the automotive industry, which limits the application of TEPs, since they are already expanded at such temperature. In this case, the use of inorganic additives, such as expandable graphite, are well suited. Their expansion temperature lies at about 200 °C (see Figure 14). Additionally, a significant advantage is the high volumetric expansion, which enables the graphite to be effective, even at low concentrations, and the possibility to be applied in tough polymeric matrices [208].

### 4.3. Final Considerations on the State of the Art about Debonding

This overview has shown different technologies known for debonding on-demand of ABJs. It is essential to realize that adhesive bonds are systems and that every change in a single aspect affects the whole system. It is necessary to find the appropriate technology, which serves all the requirements of an ABJ during its whole life cycle.

For structural adhesive bonds, complete debonding is not reachable, but a weakening of the ABJ can be enabled, so a reduced mechanical force is necessary to debond the joint.

The majority of debonding technologies involve the use of functional additives that are mixed into the adhesive (or primer layer) in quantities of up to 20 wt.%. In all those cases, the mechanical properties of the adhesive are lowered. New debonding methods must be developed for specific applications.

## 5. Critical Overview of the Connection between R&D Efforts and Industry Standards

For an effective implementation of the above described emerging technologies, the bridge between research and industry must be successful. In this section, relevant procedures and a critical overview about the connection between R&D efforts and industry standards are given, with a focus on the aerospace industry.

In the various phases of the introduction of a new adhesively bonded system, various kinds of tests are relevant. In the initial development phase, the main research is focused on producing the materials, or performing the processes, and to understand the new adhesively-bonded system. The understanding is a relevant factor for optimizing the performance, as well as maintaining the performance by identifying critical parameters and related boundaries. This latter aspect is essential for certifying the bonding process since the fabrication process requires close control to produce a consistently sound structure. In the case of aeronautics, as stated by the airworthiness authorities (e.g., European Aviation Safety Agency, EASA and Federal Aviation Administration, FAA) adhesive bonding is a special process and the following certification specification (CS) applies, according to the Code of Federal Regulations (14-CFR) Subpart C Section 25.605—Fabrication methods:

(1) “The method of fabrication used must produce a consistently sound structure. If a fabrication process (such as gluing (bonding), spot welding, or heat-treating) requires close control to reach this objective, the process must be performed under an approved process specification.”
(2) “Each new aircraft fabrication method must be substantiated by a test program.”

**Figure 15:**
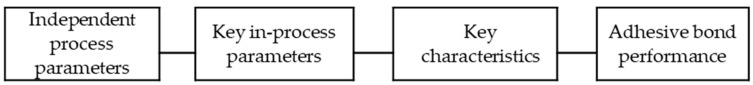
Illustration between various parameters and the final performance of a new adhesively-bonded system.

This implies, on one hand, that key process parameters and key characteristics must be known to enable close control of the various steps of the fabrication process. Additionally, a test program is required to substantiate the relations between the various parameters, as shown below in Figure 15, as well as to generate objective evidence of the reproducibility of the performance of a new adhesively-bonded system.

Basic generic technical requirements for structural adhesive bonding are:

(a) Good durable adhesion;
(b) Capability for the transfer of a (limited) load;
(c) Cohesive fracture of the adhesive (better: no adhesion fracture).

However, most suitable tests to determine the performance depend strongly on the development phase and the item to be developed. The following topics to be introduced can be identified:

(a) New substrates (mostly new metal or new composite material);
(b) New surface treatment;
(c) New bond primer;
(d) New adhesive material.

**Figure 16:**
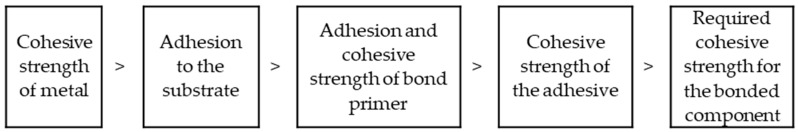
Required strength of the various elements of the adhesive bond system with structural adhesive bonding on aluminum (“>” means larger than, with the highest required strength on the left side, and the lowest required strength on the right side).

During R&D usually a great deal of attention is paid to the left side of Figure 15, which is essential for in-depth knowledge. However, one must be warned that enough destructive testing must be conducted to correlate all relations with the final performance, as shown on the right side of Figure 15. For certification the complete adhesive bond system must be tested substantially, but in the development phase only a few specific tests are essential to determine/optimize the suitability of the item in development. The selection of the most adequate tests is related to the specific strength requirements that are applicable for structural adhesive bonding, namely 100% cohesive fracture (Figure 16). Additionally, the general approach is to keep the ABJs not critical for the performance of aircraft parts.

With adhesive bonding of aluminum typically the adhesion strength and, if applicable, the bond primer strength should be somewhere between the strength of the aluminum adherend and the cohesive strength of the adhesive (Figure 16, left side). For testing adhesion with new pre-treatments, only peel tests are valid [209,210,211,212,213,214,215] and, alternatively, a wedge crack extension test [216]. When enough adhesion can be obtained, a 100% cohesive fracture will be achieved with these peel tests [121,169], and the optimum peel test values are dominated by the cohesive strength of the adhesive. Flexural properties of the thin adherend slightly affect the optimum peel test values [209,210,211,212,213,214,215]. With structural adhesive bonding of aluminum peel test values of 200–300 N/25 mm width are usual in the aerospace industry. When such strong adhesives are used for the bonding of fiber-reinforced laminates, delamination may occur in the composite’s adherends during peel testing, and softer adhesive materials might be preferred [212,213]. With 100% cohesive fracture in the peel tests the cohesive strength of the joint is solely related to the strength of the adhesive and can be determined, for example, with lap shear tests [217]. Frequently, a lap shear test is used to examine the quality of the surface treatment as well, but this test is not as sensitive for variations in adhesion. Variations in lap shear strength achieved for a certain surface treatment, are a signal of its unsuitability. With modern adhesive materials for adhesive bonding of aluminum lap shear strength values of 25–35 MPa can be expected and these values are the standard required for the introduction of new adhesives in the aerospace industry. However, the conventional aluminum-bonded designs are rather conservative to keep the ABJs not critical for the performance of aircraft parts (Figure 16, right side). Consequently, the structural performance of the adhesive bonded component is governed by the static and fatigue properties of the aluminum, and the stresses in ABJs are far from the maximum strength of the applied adhesive. For future developments, this gives possibilities to introduce more sustainable solutions by either using the full potential of these strong adhesive for weight saving, or for the introduction of alternative adhesive materials based on eco-friendly chemistries with less cohesive strength.

Various test methods have been developed to test the adhesion to the substrates, depending on the kind of substrate. This can be, for example, aluminum sheet material [209,210,214,215], honeycomb [211], composite material [212], or metal-composite hybrids [213]. The American Society for Testing and Materials (ASTM) guide for selecting aerospace and general-purpose adhesives and sealants should be consulted for specifications on the different standard test methods [218]. During the initial stage of development, it is advisable to limit to dry peel testing for screening tests [209,210,211,212,213], or wedge crack extension tests with relatively short moisture exposures [216]. Popular tests in the aerospace industry are the floating roller peel test and the wedge tests, as described in ASTM Standards D3167 [210] and D3762 [216], respectively. The floating roller peel test simulates mode I and II loading, which is suitable to evaluate the quality of the initial bonding [219]. With more promising adhesive systems it is worthwhile to continue testing in humid conditions, with wet peel testing [121,214,215] or peel testing after humidity exposure at, for example, 60 °C, >95% Relative Humidity (RH) [209,210,212,213,220], or wedge crack extension tests with humidity exposure of up to three weeks. Under wet conditions, bond durability is tested, as water ingress is often cited as the main cause for environmental adhesion failure [145]. With successful metal bonding systems it is relevant to determine, as well, the resistance to bond line corrosion with exposure of the unprotected peel specimen to salt spray exposure [209,210,221,222,223,224], and to determine if the surface treatment does not have a negative effect on the fatigue properties of the metal substrate [225]. For final qualification of a new adhesive bonding system both peel testing and lap shear testing must be conducted in various environmental conditions [220], as well as with exposure to various fluids, such as solvents, hydraulic fluids, or de-icing fluids [226].

For reproducing the quality achieved during R&D works and qualification testing, it is essential that all relevant process parameters and key characteristics and their limits are well known, in order to compile a process specification. This implies the relations between these parameters and adhesion and/or cohesive strength must be substantiated by enough testing involving parameters variation, and the resulting performance (peel or lap shear tests).

Subsequently, for industrial implementation the following actions are required:

(a) Show compliance with the process specification;
(b) Demonstrate effectivity of the implemented fabrication method in industrial environment;
(c) Demonstrate homogeneity of the process on full industrial scale:
  - Homogeneity of process baths and solutions (concentration/temperature);
  - Homogeneity of the surface characteristics over the entire working area of process baths, or realistic product dimensions. For example, with aluminum a uniform coating weight over the entire range of the anodizing baths [15];
  - Homogeneity of the adhesion over the entire working area of process baths, or realistic product dimensions;
  - Homogeneity of the bond primer characteristics and adhesion over the entire area of realistic product dimensions;
  - Homogeneity of oven or autoclaves over the entire working area with realistic products;
  - Compliance to the required bond line quality of relevant products.
(d) Demonstrate reproducibility of recurring process control tests:
  - Peel tests for multiple pre-treatment runs;
  - Lap shear test for multiple autoclave runs.

When successful, a production site can be released for recurring production with the inspections and tests as given in Table 4.

## 6. Concluding Remarks

This paper gives an overview of the latest material developments for achieving eco-friendly structural adhesives and meeting REACH compliance regulations for material and surface preparations regarding the structural application of adhesively-bonded joints. For the sake of recyclability and repair of ABJ, an overview of the existing debonding techniques available is also given. Finally, a critical overview of the process for qualifications/certification of these emerging technologies is described.

From the main topics addressed during this manuscript, the following conclusions can be drawn:

Eco-friendly structural adhesives:

- The need for eco-innovative adhesive materials stimulates the investment on efficient and sustainable production routes of new polymers from non-virgin petrochemical and bio-based raw materials, creating new “green” businesses opportunities. Alternative feedstocks, such as recycled plastics, industrial wastes—e.g., vegetable oils, sustainable biomass, and modified biopolymers like cellulose and starch—have been investigated. Their penetration in the adhesives industry are in different development stages, with cardanol-derived resins being one example of a commercially available product.
- Mechanical properties of the adhesive materials are often compromised when moving away from conventional, well established petrochemical-derived polymers, however, the incorporation of nanoparticles, or the microencapsulation of hazardous, but efficient, cross-linkers are potential strategies to achieve eco-friendly structural adhesives.

REACH-compliant surface pre-treatments:

- REACH regulations have stimulated the development of a new range of surface pre-treatments. However, the complete replacement of the hazard materials has proved to be challenging, especially when durability needs to be assured.
- Many REACH-compliant surface pre-treatments have been adopted by many sectors, however, an analogue replacement in the aerospace industry, with its high level of performance and safety, proved to be a very challenging task. In addition, the time to test and qualify new systems for aviation is much longer compared to other industries. Therefore, some Cr(VI)-based substances are still used to date to assure the required level of performance in ABJs, while many other steps of the pre-treatment process already employ environmentally-friendlier alternatives.

Debonding techniques:

- For structural adhesive bonds, complete debonding is not reachable, but a weakening of the ABJ can be enabled, so a reduced mechanical force is necessary to debond the joint.
- The majority of debonding technologies involve the use of functional additives that are mixed into the adhesive (or primer layer) in quantities of up to 20 wt%. In all those cases, the mechanical properties of the adhesive are compromised. New debonding methods must be developed for specific applications.

For successful implementation of these emerging technologies on an industrial scale, a process of qualification and or certification is needed. This process should assure the reproducibility of the quality achieved during R&D works and qualification testing upon industrial implementation. It is essential that all relevant process parameters and key characteristics and their limits are well known, in order to compile a process specification. This implies the relations between these parameters and adhesion and/or cohesive strength must be substantiated by enough testing involving parameter variation, and the resulting performance (peel or lap shear tests).

## Figures and Tables

**Table 1:** Classification of synthetic adhesives and sealants [11,12,13].

Adhesive	Properties	Applications (Adherends)
Epoxy	High strength and temperature resistance, good durability and resistance to environmental extremes, relatively low cure temperatures (for two-component formulation (2K)), easy to use, low cost.	Most materials, metals, ceramics, polymers
Acrylics	Versatile (design flexibility), high strength, fast curing, tolerates less prepared surfaces	Cloth, plastics, metals
Polyurethanes	Good flexibility at low temperatures, resistant to fatigue, impact and durability, ideal for creating strong flexible bonds between dissimilar materials.	Plastics, metals, rubber
Cyanoacrylates (superglues)	Fast bonding capability to plastic and rubber but poor to moisture and temperature	Almost any adherends
Anaerobics	Fastening and sealing without light, heat or oxygen, suitable for cylindrical shapes	Metals
Silicones	Excellent sealant for low stress applications, high flexibility, very high temperature resistance, long cure times (for one-component formulations (1K)), low strength	Metals, glass, paper, plastics, rubber, fluorocarbons
Phenolics	Good strength retention for short periods of time, limited resistance to thermal shock, low cost	Metals, wood
Polyimides	Thermal stability, dependent on a number of factors, difficult process ability, expensive	Cloth, plastics
Bismaleimides	Very rigid, low peel properties	Metals, glass, ceramics, plastics
Amino resins (e.g., urea-formaldehyde)	High strength, rigidity, cost effectiveness, and fast cure	Wood

**Table 2:** ABJs’ advantages and disadvantages [1,12,15].

Adhesively Bonded Joint Advantages	Adhesively Bonded Joint Disadvantages
Expressive weight savings, as there are no rivets (points of stress concentration)	Requires surface preparation—thorough cleaning/degreasing
More evenly distributed stresses	Curing times can be significant
Cost savings: hole fabrication is not needed	For curing, heat and pressure may be needed
Excellent fatigue resistance	Strength consistency is highly dependent on rigid process control
Increased vibration and shock resistance	Conventional techniques of non-destructive inspection of ABJs is difficult
Increased compliance to critical tolerances	Adhesive shelf-life is limited; thus, special storage conditions are required
Provides a way to seal the entire bonding area	Lower humidity and temperature resistance
Enables joining of dissimilar base materials	
Smooth contours and sections around joint areas	

**Table 3:** Peeling strength tests’ results [105].

Crosslinker Added to the OH Pre-Polymer	Average Load per Unit Width of Bond	Type of Failure Observed in the Peeling Strength Test
None	<2 N/mm	Adhesive, at the substrate/adhesive interface
IPDI	2.97 N/mm	Cohesive, through the adhesive
Microencapsulated IPDI (I MCs)	2.99 N/mm	Structural and cohesive rupture

**Table 4:** Overview of activities of close control of the various steps of the fabrication process for structural metal bonding.

Creation of Performance and Reproducibility		Validation of Process and Performance by Quality Control		
Qualified/Approved Process	Workmanship/Execution of the Process	Examples of Process Inspection	Examples of Accompanying Tests	Non-Destructive Testing
Surface pre-treatment	In pre-treatment line	Racking of metal parts, process parameters	Chemical analyses of solutions Oxide weights Peel test	Visual, and presence of oxide
Bond primer application	In contamination and humidity-controlled primer shop	Primer composition, process parameters, working method	Peel test	Primer thickness, and visual inspection
Adhesive joining	In contamination and humidity-controlled lay-up area	Adhesive quality, correct assembly, cure pressure, temperature, and time	Lap shear	Visual check of the adhesive squeeze-out; non-destructive testing for contact with bond surface, inclusions, local pressure variations, and adhesive quality (porosity)
